# Novel Skeletal Rearrangements of the Tigliane Diterpenoid Core

**DOI:** 10.1021/acs.jnatprod.3c00834

**Published:** 2023-11-22

**Authors:** Chiara Maioli, Hawraz Ibrahim M. Amin, Giuseppina Chianese, Alberto Minassi, Paul W. Reddell, Simone Gaeta, Orazio Taglialatela-Scafati, Giovanni Appendino

**Affiliations:** † Dipartimento di Scienze del Farmaco, Università degli Studi del Piemonte Orientale Amedeo Avogadro, Largo Donegani 2, 28100 Novara, Italy; ¥ Dipartimento di Farmacia, Università di Napoli Federico II, Via Montesano 49, 80131 Napoli, Italy; ‡ QBiotics Group Limited, 165, Moggill Road, 4068, Taringa, Brisbane, QLD, Australia

## Abstract

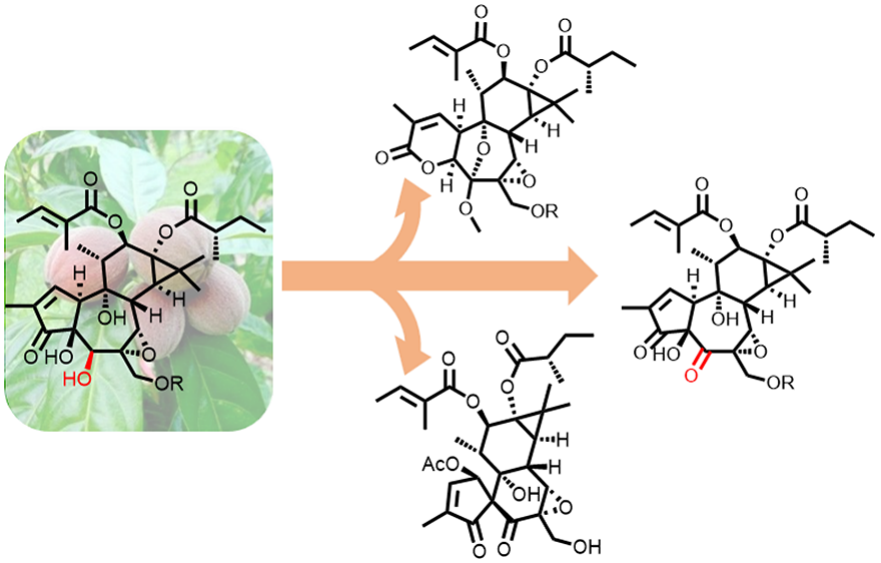

To investigate the role of the secondary 5-hydroxy group in the activity of the anticancer drug tigilanol tiglate (**2b**) (Stelfonta), oxidation of this epoxytigliane diterpenoid from the Australian rainforest plant *Fontainea picrosperma* was attempted. Eventually, 5-dehydrotigilanol tiglate (**3a**) proved too unstable to be characterized in terms of biological activity and, therefore, was not a suitable tool compound for bioactivity studies. On the other hand, a series of remarkable skeletal rearrangements associated with the presence of a 5-keto group were discovered during its synthesis, including a dismutative ring expansion of ring A and a mechanistically unprecedented dyotropic substituent swap around the C-4/C-10 bond. Taken together, these observations highlight the propensity of the α-hydroxy-β-diketone system to trigger complex skeletal rearrangements and pave the way to new areas of the natural products chemical space.

Studies spanning over half a century have clarified the basic structure–activity relationships (SARs) of phorbol (**1a**) esters. Early in vivo studies based on cancer promotion (7,12-dimethylbenzanthracene-induced formation of papilloma in mice)^1^ and irritancy (mouse ear erythema assay)^2^ highlighted the critical role played by the C-20 hydroxy and the esterification of the C-12- and C-13-hydroxy groups,^3^ with potency peaking when a long-chain acyl group is present at the secondary C-12 hydroxy and a short-chain acyl group at the tertiary C-13 hydroxy.^3^ The acyl decoration of phorbol 12-myristate-13-acetate (PMA, **1b**) exemplifies these findings,^3^ and these conclusions were confirmed by cellular studies culminating in the identification of protein kinase C (PKC), a family of serine-threonine kinases, as the major target of phorbol esters.^4,5^ These structure–activity studies were sustained by the availability of phorbol (**1a**) from croton oil, the only abundant source of this otherwise rare diterpene polyol.^6^ Paradoxically, none of the more widespread analogues of phorbol (5-deoxyphorbol, 12-deoxyphorbol, 17-hydroxyphorbol) can be obtained by isolation in amounts sufficient to sustain systematic SAR studies,^3^ while the total synthesis of phorbol has so far remained of exclusive academic relevance.^7^

This context of limited template diversification was overcome by the remarkable discovery of very large amounts of tigliane polyesters with a functionalized phorbol framework in the seed kernels of the blushwood tree (*Fontainea picrosperma* C. T. White).^8,9^ Phorboids from *F. picrosperma*, a plant endemic to the rainforest of Queensland (Australia),^10^ share a 5β-hydroxy-6,7α-epoxy phorbol core (**2a**), as exemplified by the diester **2b** (tigilanol tiglate, EBC-46). This compound was successfully developed as a topical veterinary anticancer drug (Stelfonta),^11^ and it is currently under phase II clinical study for human soft tissue sarcoma (STS) and head-and-neck malignancies (HMN).^12^

The availability of the tigliane polyol **2a** provides a unique opportunity to systematically explore point-like modifications of the pharmacophore and their possible pharmacological implications. Tigilanol tiglate (**2b**) shows a PKC activation profile significantly different from the one of PMA (**1b**), retaining activity on PKC isoforms from the conventional family (α, β1, β2, γ), but lacking affinity for those from the novel family (δ, ε, θ, η),^8,13^ a change associated with the modification of the tigliane ring B (epoxidation of the 6,7-double bond and hydroxylation at C-5)^8,13^ (Chart 1). The biological profile of phorbol esters is substantially retained in their α-epoxides,^13^ but nothing is known on the effect of the occurrence of the hydroxy group at C-5. This functionality, acting as a hydrogen-bonding donor/acceptor, has the potential to perturb the delicate network of hydrogen bondings involving the oxygenated functions at C-20, a critical element for binding of phorbol esters to PKC.^14^ Previous attempts at direct allylic oxidation of the tigliane scaffold highlighted poor accessibility of the methylenic position at C-5, leading to oxidative fragmentations of the diterpenoid.^15^ Therefore, the preparation of 5-dehydro tigilanol tiglate(**3a**) (Chart 1) was perceived as critical for the study of the structure–activity relationships of epoxytiglianes.

**Chart 1:**
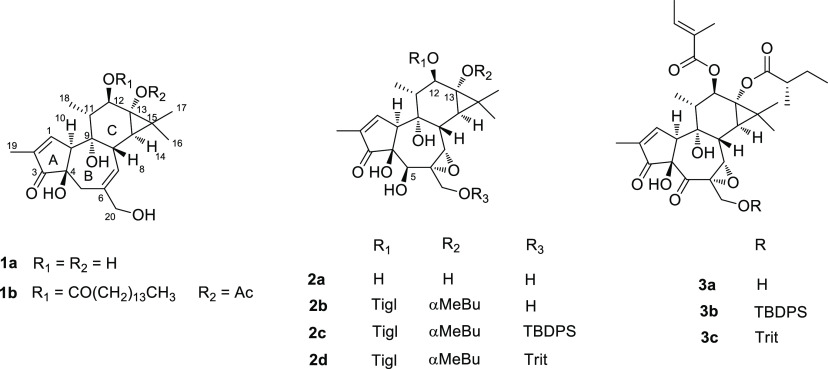


## Results and Discussion

The direct oxidation of tigilanol tigliate (**2b**) to its 5-dehydro derivative was first investigated. Various reagents have been developed for the chemoselective oxidation of secondary alcohols in the presence of primary ones, but these are, in general, incompatible with the presence of double bonds.^16^ On the other hand, examples of chemoselective secondary alcohol oxidation have been reported with periodinanes,^15^ and, indeed, the treatment of **2b** with SIBX (stabilized iodoxybenzoic acid) afforded a reaction mixture containing its 5-dehydro derivative **3a**. However, the yield was very low, and the purification from the reaction mixture was problematic in light also of the instability of the compound (vide infra). Therefore, an alternative three-step strategy based on protection of the C-20 hydroxy, oxidation of the C-5 alcohol, and removal of the protecting group was pursued.

**Figure 1:**
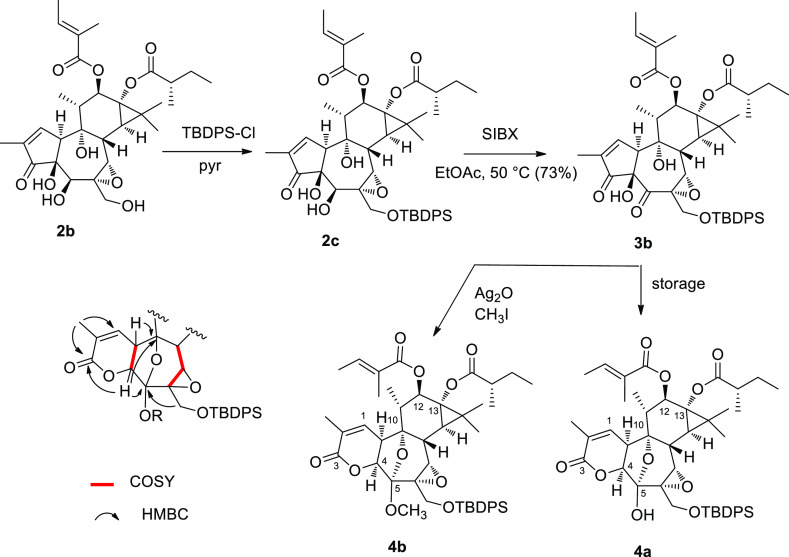
Formation of the rearranged product **4a** and its methylated derivative **4b** from tigilanol tiglate **2b**. Bottom left: key COSY and HMBC correlations used to deduce their structures.

The primary hydroxy group at C-20 of tigilanol tigliate could be chemoselectively silylated [*tert*-butyldiphenylsilyl chloride (TBDPS-Cl)-imidazole] or tritylated (trityl chloride/pyridine), affording, respectively, compounds **2c** and **2d**, which are protected with orthogonally cleavable groups (mildly basic fluoridolysis for the silyl group of **2c**, acidic conditions for the trityl group of **2d**) (Figure 1).

Oxidation of the 20-silyl derivative **2c** with SIBX afforded the 5-dehydro derivative **3b** as an interconverting mixture of carbonyl- and δ-lactol tautomers. Under storage or the mildly basic conditions of fluoridolysis, **3b** cleanly rearranged to the δ-lactone **4a** (Figure 1).

The most significant changes in the ^1^H NMR spectrum of **4a**, compared to its precursor, were the marked relative shielding of the H-1 signal (from δ_H_ 7.70 to 6.18) and the presence of a doublet at δ_H_ 4.85 (H-4), coupled to H-10. The ^13^C NMR spectrum of **4a** showed the evident shielding of C-1 (from δ_C_ 164 to 136.2) and of C-3 (from δ_C_ 209 to 161.3) and the presence of an acetal carbon at δ_C_ 103.3. The combined analysis of 2D NMR COSY, HSQC, and HMBC spectra provided evidence to deduce the structure of **4a**. Key COSY and HMBC correlations detected for rings A and B are shown in Figure 1. The strong NOESY cross-peak H-4/H-10 indicated the configuration at the C-4 stereocenter.

**Figure 2:**
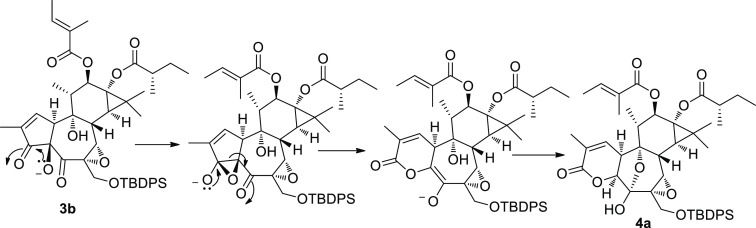
Possible mechanism for the rearrangement affording δ-lactone **4a**.

The δ-lactone **4a** seemingly derives from a post-oxidative base-induced dismutative process involving the formation of a 3,4-epoxide, which next undergoes fragmentation of the C-3–C-4 bond with ring expansion and generation of a 5-enol. After tautomerization, the resulting 5-ketone is trapped by the tertiary 9-hydroxy to afford a hemiketal (Figure 2). Remarkably, the 6,7-epoxide group remained unscathed in the reaction. A literature search showed that this mechanistically surprising rearrangement had already been documented in other polycyclic α-hydroxy-β-diketones,^17−19^ suggesting its generality.

**Figure 3:**
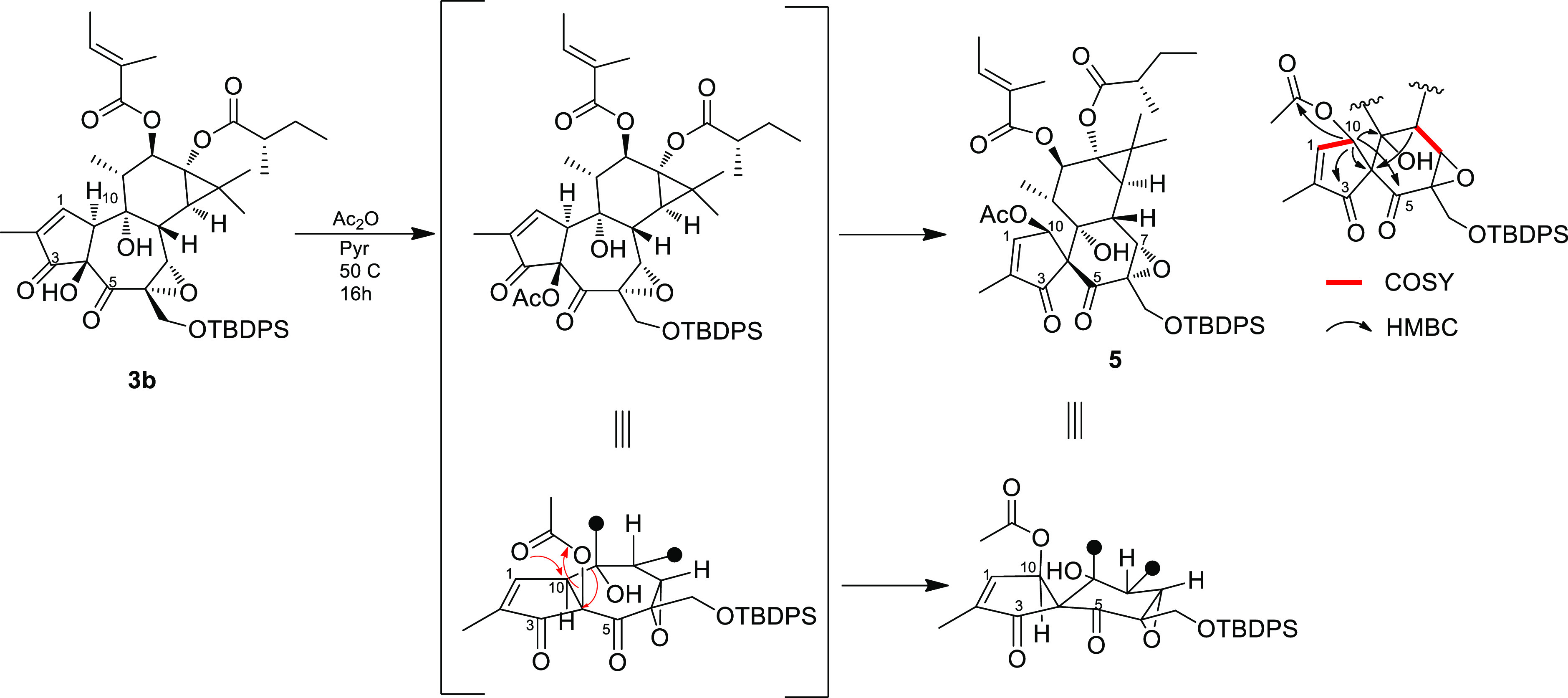
Possible mechanism for the rearrangement of **3b** into the spiro-enone **5**. Right: COSY and key HMBC correlations of rings A and B of **5**.

Puzzled and intrigued by the dependence of the tautomeric equilibrium of the 5-dehydro derivatives from the nature of the group bound at the 20-hydroxy, we tried to trap the lactol tautomer of the 20-silyl derivative **3b** as a methyl derivative, but the basic conditions of the reaction (Ag_2_O, MeI) afforded the methyl acetal of the rearranged lactone, **4b** (Figure 1). Conversely, attempts to trap the lactol tautomer of **3b** as an acetate triggered a complex novel rearrangement that afforded the spiro-enone **5** as the major reaction product (Figure 3).

The ^1^H NMR spectrum of **5** was analyzed with the help of the 2D NMR COSY spectrum and revealed that, while ring C and its appended groups must be unscathed, dramatic structural changes have happened at rings A/B. The most evident change was the vicinal coupling of H-1 (δ_H_ 7.41) with a broad singlet at δ_H_ 5.51. Having associated all of the proton signals with the directly linked carbon atoms, we could determine that H-10 must be bound to an oxygenated carbon (δ_C_ 74.4). The HMBC cross-peak of H-10 with the acetate ester carbonyl unambiguously indicated that acetylation had occurred at C-10. The signal of H-10 showed a network of HMBC correlations (Figure 3), including those with two ketone carbonyls, with oxygenated C-9 and with a quaternary carbon resonating at δ_C_ 64.1. The spiro nature of this latter carbon was evidenced by its crucial correlation with H-8.

The formation of compound **5** could be the result of the acetylation of the C-4 hydroxy group of **3b**, followed by a type I dyotropic rearrangement along the C-4/C-10 bond of C-9 (from C-10 to C-4) and of the acetate group (from C-4 to C-10) (Figure 3), with overall inversion of configuration of C-4 and C-10. Cross-peaks of the NOESY spectrum of **5** confirmed the configurational arrangement predicted by this mechanism (NOE contact of H-10 with H-11 and H_3_-18). Dyotropic rearrangements are very rare and have been mostly reported in sterically congested polycyclic frameworks, where strain relief is the primary driving force.^20^ In our case, the reaction is seemingly triggered by the inherent instability of the α-hydroxy-β-dicarbonyl system, which features a partial positive charge on three adjacent carbons.

To avoid the pitfall of the basic deprotection, the SIBX oxidation was next carried out on the trityl derivative **2d**, affording the 20-trityl derivative **3c**, again as a mixture of a carbonyl and a hemiketal tautomers. Acidic deprotection was uneventful and cleanly provided 5-dehydro tigilanol tigliate (**3a**) as a single carbonyl tautomer. However, during the purification and the spectroscopic characterization of **3a**, it soon became evident that this compound is highly unstable. Compound **3a** could be stored as a frozen DMSO or benzene solution, but much to our regret, was unstable under the cellular or enzyme assay conditions, and therefore not a suitable tool compound for bioactivity studies.^21^ No specific degradation product could be isolated, but it does not seem unrealistic to assume that rearrangements similar to those observed in the 20-protected derivatives could take place, compounded by the presence of a free 20-hydroxy.

In summary, due to unforeseen instability issues associated with the presence of a carbonyl function at C-5, our attempts to shed light on the role of the C-5 hydroxy group in the activity of epoxytiglianes have substantially failed. We have, nevertheless, discovered some fascinating aspects of the chemistry of tigliane derivatives, describing diterpenoid skeleta “programmed” by the reactivity associated with the presence of an oxygen function at C-5, and whose occurrence in Nature might therefore have been anticipated by our study.

## Experimental Section

### General Experimental Procedures

Optical rotations (CHCl_3_ and MeOH) were measured at 589 nm on a P2000 (JASCO Europe s.r.l.) polarimeter. ^1^H (600 and 700 MHz) and ^13^C (150 and 175 MHz) NMR spectra were measured on a Varian spectrometer. Chemical shifts are referenced to the residual solvent signal (CDCl_3_: δ_H_ 7.26, δ_C_ 77.0). Homonuclear ^1^H connectivities were determined by the COSY experiments. Through-space ^1^H connectivities were evidenced by using a NOESY experiment with a mixing time of 300 ms. One-bond heteronuclear ^1^H–^13^C connectivities were determined by the HSQC experiment; two- and three-bond ^1^H–^13^C connectivities by gradient-HMBC experiments optimized for a ^2,3^*J* of 8 Hz. HR-ESIMS experiments were performed on an LTQ-Orbitrap mass spectrometer equipped with an ESI interface and Excalibur data system. Chemicals were purchased from Fluorochem, TCI, or Alfa Aesar and used without any further purification. Solvents were purified by distillation and dried according to the standard methods. Thin-layer chromatography was performed with 0.2 mm precoated aluminum TLC silica gel 60 F_254_ (Merck). Column chromatography was carried out using Merck silica gel 60 (0.063–0.200 mm). Tigilanol tiglate (**2b**) was supplied by INDENA s.p.a. or QBiotics Group Limited.

### Protection of the 20-Hydroxy of Tigilanol Tiglate (**2b**)

(a) As a silyl derivative: to a solution of **2b** (1.0 g, 1.77 mmol) in pyridine (10 mL) was added under stirring at room temperature (rt) *tert*-butyldiphenylsilyl chloride (1 M in CH_2_Cl_2_, 5 mL, 3 molar equiv). The reaction was monitored by TLC until completion. The mixture was next diluted with 2 M H_2_SO_4_ and 10% Na_2_SO_4_ (1:1 ratio) and extracted with EtOAc. The combined organic phases were dried over Na_2_SO_4_, filtered, and evaporated. The course of the reaction was followed by TLC (petroleum ether/EtOAc, 4:6, *R_f_*_(**2c**)_ = 0.46). The residue was purified by gravity column chromatography on silica gel (petroleum ether/EtOAc, 8:2) to afford **2c** (1.44 g, approximately quantitative) as a white amorphous powder. (b) As a trityl derivative: to a stirred solution of **2b** (50 mg, 0.089 mmol) in CH_2_Cl_2_ (2 mL) were added at rt triphenylmethyl chloride (124 mg, 0.44 mmol, 5 equiv), diisopropylethylamine (DIPEA, 57.3 mg, 0.44 mmol, 5 molar equiv), and a catalytic amount of 4-dimethylaminopyridine (DMAP). The course of the reaction was followed by TLC (petroleum ether/EtOAc, 4:6, *Rf*_(**2d**)_ = 0.88). After 16 h, the reaction mixture was diluted with brine and extracted with EtOAc. The crude product was purified by gravity column chromatography (petroleum ether/ethyl acetate, 8:2), affording **2d** (50.0 mg, 0.062 mmol, 70%) as an amorphous pale yellow powder.

#### 20-*tert*-Butydiphenylsilyl tigilanol tiglate (**2c**):

white amorphous solid; ^1^H NMR (400 MHz, CDCl_3_) δ_H_ 7.74–7.66 (m, 5H, phenyl-20-*O-*TBDPS), 7.45–7.34 (m, 6H, H-1 and phenyl-20-*O-*TBDPS), 6.83 (qq, *J* = 7.5, 1.6 Hz, 1H, H-3′), 5.45 (d, *J* = 9.9 Hz, 1H, H-12), 4.41 (s, 1H, H-5), 4.26 (d, *J* = 11.1 Hz, 1H, H-20a), 4.21 (t, *J* = 2.8 Hz, 1H, H-10), 3.46 (d, *J* = 11.1 Hz, 1H, H-20b), 3.14 (d, *J* = 6.5 Hz, 1H, H-8), 3.10 (s, 1H, H-7), 2.39 (m, *J* = 6.9 Hz, 1H, H-2″), 1.97 (dq, *J* = 9.9, 6.4 Hz, 1H, H-11), 1.82 (d, *J* = 1.6 Hz, 3H, H-5′), 1.80 (d, *J* = 1.2 Hz, 3H, H-2′), 1.78 (bd, *J* = 1.3 Hz, 3H, H-19), 1.73 (m, *J* = 14.0, 7.1 Hz, 1H, H-3″a), 1.46 (dt, *J* = 14.0, 7.1 Hz, 1H, H-3″b), 1.23 (s, 3H, H-17), 1.19 (s, 3H, H-16), 1.14 (d, *J* = 6.5 Hz, 1H, H-14), 1.04 (s, 9H, *tert*-butyl in 20-O-TBDPS moiety), 0.94 (t, *J* = 7.4 Hz, 3H, H-4″), 0.87 (d, *J* = 6.4 Hz, 3H, H-18); ^13^C NMR (100 MHz, CDCl_3_) δ_C_ 209.7 (C-3), 178.9 (C-1″), 167.6 (C-1′), 164.3 (C-1), 137.6 (C-3′), 135.7 (Ph in 20-*O*-TBDPS), 135.7 (Ph in 20-*O*-TBDPS), 133.5 (C-2), 133.3 (Ph in 20-*O*-TBDPS), 133.2 (Ph in 20-*O*-TBDPS), 129.9 (Ph in 20-*O*-TBDPS), 129.8 (Ph in 20-*O*-TBDPS), 128.6 (C-2′), 127.9 (Ph in 20-*O*-TBDPS), 127.9 (Ph in 20-*O*-TBDPS), 77.4 (C-9), 76.9 (C-12), 72.8 (C-4), 69.6 (C-7), 66.76 (C-5), 65.7 (C-20), 65.0 (C-13), 63.0 (C-6), 48.9 (C-10), 46.1 (C-11), 41.3 (C-2″), 36.4 (C-8), 35.9 (Cq *tert*-butyl in 20-*O*-TBDPS moiety), 26.9 (*tert*-butyl in 20-*O*-TBDPS moiety), 26.7 (C-15), 26.3 (C-3″), 23.8 (C-17), 17.4 (C-16), 16.3 (C-5″), 15.2 (C-18), 14.6 (C-4′), 12.4 (C-5′), 11.7 (C-4″), 10.0 (C-19); HRESIMS *m*/*z* 822.3777 [M + Na]^+^ (calcd for C_46_H_59_O_10_SiNa, 822.3775).

#### 20-Trityl tigilanoltiglate (**2d**):

pale yellow amorphous solid; ^1^H NMR (400 MHz, CDCl_3_) δ_H_ 7.75 (q, *J* = 1.2 Hz, 1H, H-1), 7.55–7.45 (m, 15H, 20-*O*-Trit), 7.38–7.20 (m, 10H, 20-*O*-Trit), 6.83 (qq, *J* = 7.5, 1.6 Hz, 1H, H-3′), 6.02 (s, 1H, OH), 5.47 (d, *J* = 9.9 Hz, 1H, H-12), 4.55 (s, 1H, H-5), 4.30 (d, *J* = 1.2 Hz, 1H, H-10), 3.70 (d, *J* = 1.0 Hz, 1H, OH), 3.64 (d, *J* = 9.9 Hz, 1H, H-20a), 3.41 (d, *J* = 4.2 Hz, 1H, OH), 3.14 (d, *J* = 6.5 Hz, 1H, H-8), 2.95 (d, *J* = 9.9 Hz, 1H, H-20b), 2.42 (q, *J* = 6.9 Hz, 1H, H-2″), 1.98 (dq, *J* = 10.0, 6.4 Hz, 1H, H-11), 1.83 (d, *J* = 1.4 Hz, 3H, H-5′), 1.82–1.80 (m, 3H, H-4′), 1.79 (d, *J* = 1.2 Hz, 1H, H-19), 1.78–1.71 (m, 1H, H-3″a), 1.49 (dt, *J* = 14.0, 7.1 Hz, 1H, H-3″b), 1.25 (d, *J* = 6.7 Hz, 1H, H-14), 1.23 (s, 3H, H-17), 1.21 (s, 3H, H-16), 1.16 (d, *J* = 7.0 Hz, 3H, H-5″), 0.97 (t, *J* = 7.4 Hz, 3H, H-4″), 0.89 (d, *J* = 6.5 Hz, 3H, H-18); ^13^C NMR (100 MHz, CDCl_3_) δ_C_ 209.3 (C-3), 179.0 (C-1″), 167.6 (C-1′), 163.9 (C-1), 143.8 (Ph in 20-*O*-Trit), 137.7 (C-3′), 133.6 (C-2), 128.8 (Ph in 20-*O*-Trit), 128.6 (C-2′), 128.0 (Ph in 20-*O*-Trit), 127.2 (Ph in 20-*O*-Trit), 86.6 (Cq in 20-*O*-Trit), 77.4 (C-9), 72.91 (C-12), 68.9 (C-4), 66.7 (C-5), 66.0 (C-13), 65.7 (C-20), 65.4 (C-7), 62.7 (C-6), 48.9 (C-10), 45.9 (C-11), 41.3 (C-2″), 36.4 (C-14), 35.8 (C-8), 26.6 (C-15), 26.3 (C-3″), 23.8 (C-17), 17.3 (C-16), 16.3 (C-5″), 15.1 (C-18), 14.5 (C-4′), 12.3 (C-5′), 11.7 (C-4″), 10.0 (C-19); HRESIMS *m*/*z*826.3699 [M + Na]^+^ (calcd for C_49_H_55_O_10_Na, 826.3693).

### SIBX Oxidation

Oxidation of **2b** as exemplificative: To a solution of **2b** (1 equiv) in EtOAc (1:10 v/v) was added SIBX (3.3 equiv) at rt. The reaction was stirred overnight at 50 °C and monitored by TLC until completion. The mixture was worked up by filtration on Celite, and the cake washed with EtOAc. The filtrate was diluted with an aqueous saturated solution of Na_2_S_2_O_3_ and extracted with EtOAc. The combined organic phases were dried over Na_2_SO_4_, filtered, and evaporated. The residue was purified by gravity column chromatography on silica gel (petroleum ether/EtOAc, 6:4), affording **3a** as an unstable, white, amorphous powder. When the reaction was carried out on **2c** and **2d**, compounds **3b** and **3c** were respectively obtained (**3b**, 73%; **3c**, 40%). Under storage, compound **3b** cleanly converted into lactone **4a**.

### Acidic Deprotection of **3c**

To a solution of **3c** (30 mg, 0.037 mmol) in 1.1 mL of MeCN/H_2_O (10:1) was added 56 μL of an aqueous solution of 0.5 M HClO_4_ at rt. The reaction was stirred for 16 h, monitoring its course by TLC (petroleum ether/EtOAc, 6:4, *R_f_*_(**3c**)_ = 0.81; *R_f_*_(**3a**)_ = 0.12). The reaction mixture was quenched with 400 μL of a 10% aqueous solution of NaOAc, diluted with brine, and extracted with EtOAc. The combined organic phases were dried over Na_2_SO_4_, filtered, and evaporated. The residue was purified by gravity column chromatography on silica gel (petroleum ether/EtOAc, 6:4), affording **3a** (11.2 mg, 0.02 mmol, 54%) as a white, amorphous powder.

#### 5-Dehydrotigilanol tiglate (**3a**):

white amorphous powder; ^1^H NMR (400 MHz, CDCl_3_) δ_H_ 7.79–7.63 (m, 1H, H-1), 6.86–6.76 (m, 1H, H-3′), 6.20 (br s, 1H, OH), 5.49 (d, *J* = 10.1 Hz, 1H, H-12), 5.09 (t, *J* = 2.8 Hz, 1H, H-10), 4.13 (d, *J* = 12.8 Hz, 1H, H-20a), 3.87 (d, *J* = 12.9 Hz, 1H, H-20b), 3.58 (d, *J* = 6.5 Hz, 1H, H-8), 2.84 (d, *J* = 5.9 Hz, 1H, H-7), 2.45–2.36 (m, 1H, H-2″), 2.02–1.96 (m, 1H, H-11), 1.82 (d, *J* = 1.01 Hz, 3H, H-5′), 1.79 (dd, *J* = 7.1, 1.2 Hz, 3H, H-4′), 1.77 (dd, *J* = 2.9, 1.3 Hz, 3H, H-19), 1.72 (d, *J* = 7.1 Hz, 1H, H-3″a), 1.52–1.43 (m, 1H, H-3″b), 1.26 (s, 3H, H-17), 1.21 (s, 3H, H-16), 1.15 (d, *J* = 6.8 Hz, 3H, H-5″), 1.07 (d, *J* = 6.5 Hz, 1H, H-14), 0.95 (t, *J* = Hz, 3H, H-4″), 0.86 (d, *J* = 6.5 Hz, 3H, H-18); ^13^C NMR (100 MHz, CDCl_3_) δ_C_ 204.73 (C-3), 201.52 (C-5), 179.26 (C-1″), 167.55 (C-1′), 161.04 (C-1), 137.91 (C-3′), 133.05 (C-2), 128.51 (C-2′), 77.57 (C-9), 76.02 (C-12), 75.86 (C-4), 65.41 (C-13), 65.26 (C-7), 65.12 (C-20), 60.96 (C-6), 49.51(C-10), 45.88 (C-11), 41.34 (C-2″), 36.45 (C-14), 36.26 (C-8), 26.75 (C-15), 26.31 (C-3″), 23.83 (C-17), 17.06 (C-16), 16.30 (C-5″), 14.60 (C-18), 14.55 (C-4′), 12.40 (C-5′), 11.76 (C-4″), 10.30 (C-19); HRESIMS *m*/*z* 583.2522 [M + Na]^+^ (calcd for C_30_H_40_O_10_Na, *m*/*z* 583.2519).

#### 20-*tert*-Butyldiphenylsilyl 3,4-*seco*,3,4-oxa-5-dehydrotigilanol tiglate 9,5 hemiketal (**4a**):

colorless oil; [α]^20^_D_ −2 (*c* 0.1, CHCl_3_); ^1^H NMR (400 MHz, CDCl_3_) δ_H_ 7.73–7.46 (10H, m, *O*-TBDPS), 6.18 (1H, d, *J* = 2.6 Hz, H-1), 6.82 (1H, qq, *J* = 7.5, 1.6 Hz, H-3′), 5.77 (1H, d, *J* = 9.9 Hz, H-12), 4.85 (1H, d, *J* = 11.9 Hz, H-4), 4.41 (1H, d, *J* = 12.1 Hz, H-20a), 3.78 (1H, d, *J* = 12.1 Hz, H-20b), 3.38 (1H, d, *J* = 4.4 Hz, H-7), 3.31 (1H, m, H-10), 2.36 (1H, sxt, *J* = 6.9 Hz, H-2″), 1.97 (3H, d, *J* = 2.6 Hz, H-19), 1.90 (1H, m, H-8), 1.86 (3H, s, H-5′), 1.80 (3H, d, *J* = 7.0 Hz, H-4′), 1.72 (1H, m, H-11), 1.66 (1H, m, H-3″b), 1.42 (1H, overlapped, H-15), 1.37 (1H, m, H-3″a), 1.33 (3H, s, H-17), 1.19 (3H, s, H-16), 1.06 (9H, s, *tert*-butyl in 20-*O*-TBDPS moiety), 1.02 (3H, d, *J* = 6.9 Hz, H-18), 0.88 (3H, t, *J* = 7.4 Hz, H-4″); ^13^C NMR (CDCl_3_, 125 MHz) δ_C_ 176.3 (C-1″), 167.7 (C-1′), 161.3 (C-3), 136.7 (C-3′), 136.2 (C-1), 135.7–127.9 (diphenyl in 20-*O*-TBDPS moiety), 128.8 (C-2′), 126.7 (C-2),103.3 (C-5), 85.9 (C-9), 83.7 (C-4), 75.4 (C-12), 64.6 (C-13), 64.5 (C-20), 60.9 (C-6), 57.4 (C-7), 40.7 (C-11), 40.6 (C-2″), 37.7 (C-10), 31.7 (C-8), 30.1 (C-14), 26.7 (*tert*-butyl in 20-*O*-TBDPS moiety), 26.4 (C-3″), 26.3 (C-15), 25.0 (C-16), 18.0 (C-5′), 17.9 (C-19), 16.5 (C-17), 16.2 (C-5′′), 14.4 (C-4′), 11.7 (C-4′′), 11.6 (C-18); HRESIMS *m*/*z* 821.3697 [M + Na]^+^ (calcd for C_46_H_58_O_10_SiNa, *m*/*z*821.3698).

### Methylation of **3b**

To a solution of **3b** (110 mg, 0.137 mmol) in 1 mL of CH_2_Cl_2_ were added 43 mg of Ag_2_O (0.185 mmol, 1.35 equiv) and 85 μL of methyl iodide (1.37 mmol, 10 equiv). The reaction was stirred 3 h at rt in the dark, with monitoring of the reaction course by TLC. The reaction mixture was filtered on Celite, and the cake was washed with CH_2_Cl_2_. The filtrate was dried over Na_2_SO_4_, filtered, and evaporated. The residue was purified by gravity column chromatography on silica gel (petroleum ether/EtOAc, 9:1), affording **4b** (11 mg, 0.0137 mmol, 10%).

#### Methyl 20-*tert*-Butyldiphenylsilyl-3,4-*seco*,3,4-oxa-5-dehydrotigilanol tiglate 9→5 acetal (**4b**):

colorless oil; [α]^20^_D_ −9 (*c* 0.1, CHCl_3_); ^1^H NMR (400 MHz, CDCl_3_) δ_H_ 7.73–7.46 (10H, m, diphenyl in 20-*O*-TBDPS moiety), 6.18 (1H, d, *J* = 2.6 Hz, H-1), 6.82 (1H, qq, *J* = 7.5, 1.6 Hz, H-3′), 5.77 (1H, d, *J* = 9.9 Hz, H-12), 4.85 (1H, d, *J* = 11.9 Hz, H-4), 4.41 (1H, d, *J* = 12.1 Hz, H-20a), 3.78 (1H, d, *J* = 12.1 Hz, H-20b), 3.47 (3H, s, 5-OMe), 3.38 (1H, d, *J* = 4.4 Hz, H-7), 3.31 (1H, m, H-10), 2.36 (1H, sxt, *J* = 6.9 Hz, H-2″), 1.97 (3H, d, *J* = 2.6 Hz, H-19), 1.90 (1H, m, H-8), 1.86 (3H, s, H-5′), 1.80 (3H, d, J = 7 Hz, H-4′), 1.72 (1H, m, H-11), 1.66 (1H, m, H-3″b), 1.42 (1H, overlapped, H-15), 1.37 (1H, m, H-3″a), 1.33 (3H, s, H-17), 1.19 (3H, s, H-16), 1.06 (9H, s, *tert*-butyl in 20-*O*-TBDPS moiety), 1.02 (3H, d, *J* = 6.9 Hz, H-18), 0.88 (3H, t, *J* = 7.4 Hz, H-4″); ^13^C NMR (CDCl_3_, 125 MHz) δ_C_ 176.3 (C-1″), 167.7 (C-1′), 161.3 (C-3), 136.7 (C-3′), 136.2 (C-1), 135.7–127.9 (diphenyl in 20-*O*-TBDPS moiety), 128.8 (C-2′), 126.7 (C-2), 105.3 (C-5), 85.9 (C-9), 83.7 (C-4), 75.4 (C-12), 64.6 (C-13), 64.5 (C-20), 60.9 (C-6), 57.4 (C-7), 49.7 (5-OMe), 40.7 (C-11), 40.6 (C-2″), 37.7 (C-10), 31.7 (C-8), 30.1 (C-14), 26.7 (*tert*-butyl in 20-*O*-TBDPS moiety), 26.4 (C-3″), 26.3 (C-15), 25.0 (C-16), 18.0 (C-5′), 17.9 (C-19), 16.5 (C-17), 16.2 (C-5′′), 14.4 (C-4′), 11.7 (C-4′′), 11.6 (C-18); HRESIMS *m*/*z* 835.3850 [M + Na]^+^ (calcd for C_47_H_60_O_10_SiNa, 835.3853).

### Formation of Compound **5**

To a solution of **3b** (65 mg, 0.081 mmol) in 1 mL of pyridine were added 1 mL of acetic anhydride (2.268 mmol, 28 equiv) and DMAP (catalytic amount) at rt. The reaction was stirred overnight at 50 °C, with its course monitored by TLC. The mixture was quenched with MeOH, diluted with 2 M H_2_SO_4_ and 10% Na_2_SO_4_ (4 mL, 1:1 ratio), and extracted with EtOAc. The combined organic phases were dried over Na_2_SO_4_, filtered, and evaporated. The residue was purified by gravity column chromatography on silica gel (petroleum ether/EtOAc, 9:1), affording compound **5** (23.2 mg, 0.0288 mmol, 35%).

#### Compound **5**:

colorless oil; [α]^20^_D_ −26 (*c* 0.2, CHCl_3_); ^1^H NMR (600 MHz, CDCl_3_) δ_H_ 7.71–7.38 (10H, m, diphenyl in 20-*O*-TBDPS moiety), 7.41 (1H, overlapped, H-1), 6.85 (1H, qq, *J* = 7.5, 1.6 Hz, H-3′), 5.51 (1H, bs, H-10), 5.48 (1H, d, *J* = 9.8 Hz, H-12), 4.33 (1H, d, *J* = 12.1 Hz, H-20a), 3.94 (1H, d, *J* = 12.1 Hz, H-20b), 3.83 (1H, s, H-7), 2.36 (1H, sxt, *J* = 6.9 Hz, H-2″), 2.07 (3H, s, H-2‴), 2.06 (1H, overlapped, H-8), 1.85 (3H, s, H-5′), 1.84 (3H, bs, H-19), 1.83 (1H, m, H-11), 1.82 (3H, d, *J* = 7 Hz, H-4′), 1.76 (1H, m, H-3″b), 1.45 (1H, m, H-3″a), 1.45 (1H, overlapped, H-15), 1.39 (3H, s, H-17), 1.31 (3H, s, H-16), 1.05 (9H, s, *tert*-butyl in 20-*O*-TBDPS moiety), 0.95 (3H, t, *J* = 7.4 Hz, H-4″), 0.73 (3H, d, *J* = 6.9 Hz, H-18); ^13^C NMR (CDCl_3_, 150 MHz) δ_C_ 202.1 (C-5), 199.0 (C-3), 176.3 (C-1″), 169.9 (C-1‴), 167.7 (C-1′), 157.4 (C-1), 140.2 (C-2), 136.7 (C-3′), 135.7–127.9 (diphenyl in 20-*O*-TBDPS moiety), 128.8 (C-2′), 76.4 (C-12), 76.2 (C-9), 74.4 (C-10), 64.6 (C-13), 64.1 (C-4), 61.0 (C-6), 58.5 (C-7), 58.4 (C-20), 43.5 (C-11), 40.6 (C-2″), 35.9 (C-8), 32.2 (C-14), 26.7 (*tert*-butyl in 20-*O*-TBDPS moiety), 26.4 (C-3″), 26.3 (C-15), 23.8 (C-16), 20.4 (C-2‴), 18.0 (C-5′), 16.8 (C-17), 16.2 (C-5′′), 14.4 (C-4′), 13.8 (C-18), 11.7 (C-4′′), 10.5 (C-19); HRESIMS *m*/*z* 863.3813 [M + Na]^+^ (calcd for C_48_H_60_O_11_SiNa, 863.3803).

## Supporting Information Available

1D and 2D NMR spectra of selected products (PDF)
Raw NMR data files (ZIP)

## References

[ref1] Berenblum I. Cancer Res. 1941, 1, 44–50.

[ref2] Tubaro A.; Dri P.; Delbello G.; Zilli C.; Della Loggia R. Agents Actions 1986, 17, 347–349. 10.1007/BF01982641. 3962781

[ref3] Hecker E. Cancer Res. 1968, 28, 2338–2349. 5723975

[ref4] Nishizuka Y. Nature 1984, 308 (1984), 693–698. 10.1038/308693a0. 6232463

[ref5] Blumberg P. M. Cancer Res. 1988, 48, 1–8. 3275491

[ref6] Pagani A.; Gaeta S.; Savchenko A. I.; Williams C. M.; Appendino G. Beilstein J. Org. Chem. 2017, 13, 1361–1367. 10.3762/bjoc.13.133. 28781702 PMC5530722

[ref7] Liu Z.; Ding Z.; Chen K.; Xu M.; Yu T.; Tong G.; Zhang H.; Li P. Nat. Prod. Rep. 2021, 38, 1589–1617. 10.1039/D0NP00086H. 33508045

[ref8] Cullen J. K.; Boyle G. M.; Yap P.-Y.; Elmlinger S.; Simmons J. L.; Broit N.; Johns J.; Ferguson B.; Maslovskaya L. A.; Savchenko A. I.; Malek P.; Mirzayans P. M.; Porzelle A.; Bernhardt P. V.; Gordon V. A.; Reddel P. A.; Pagani A.; Appendino G.; Parsons P. G.; Williams C. M. Sci. Rep. 2021, 11, 207 10.1038/s41598-020-80397-9. 33420238 PMC7794351

[ref9] Chianese G.; Amin H. I. M.; Maioli C.; Reddell P.; Parsons P.; Cullen J.; Johns J.; Handoko H.; Boyle G.; Appendino G.; Taglialatela-Scafati O.; Gaeta S. J. Nat. Prod. 2022, 85, 1959–1966. 10.1021/acs.jnatprod.2c00226. 35973043 PMC9425429

[ref10] Grant E. L.; Conroy G. C.; Lamont R. W.; Reddell P. W.; Wallace H. M.; Ogbourne S. M. Heredity (Edinb). 2019, 123, 503–516. 10.1038/s41437-019-0231-1. 31076650 PMC6781113

[ref11] ahttps://www.ema.europa.eu/en/medicines/veterinary/EPAR/stelfonta (for EMA approval).

[ref12] https://clinicaltrials.gov/search?term=tigilanol%20tiglate.

[ref13] Wender P. A.; Buschmann N.; Cardin N. B.; Jones L. R.; Kan C.; Kee J. M.; Kowalski J. A.; Longcore K. E. Nat. Chem. 2011, 3, 615 10.1038/nchem.1074. 21778981 PMC3144521

[ref14] Katti S. S.; Krieger I. V.; Ann J.; Lee J.; Sacchettini J. C.; Igumenova T. I. NatCommun. 2022, 13, 2695 10.1038/s41467-022-30389-2. PMC9110374 35577811

[ref15] Amin H. I. M.; Maioli C.; Chianese G.; Appendino G.; Gaeta S.; Taglialatela-Scafati O. Fitoterapia 2021, 148, 104802 10.1016/j.fitote.2020.104802. 33309651

[ref16] Arterburn J. B. Tetrahedron 2001, 57, 9765–9788. 10.1016/S0040-4020(01)01009-2.

[ref17] Kawamura M.; Kamo S.; Azuma S.; Kubo K.; Sasamori T.; Tokitoh N.; Kuramochi K.; Tsubaki K. Org. Lett. 2017, 19, 301–303. 10.1021/acs.orglett.6b03541. 28044443

[ref18] zurBonsen A. B.; Peralta R. A.; Fallon T.; Huang D. M.; George J. H. Angew. Chem., Int. Ed. 2022, 61, e202203311 10.1002/anie.202203311. PMC9541541 35680561

[ref19] Kieslich D.; Christoffers J. Org. Lett. 2021, 23, 953–957. 10.1021/acs.orglett.0c04157. 33464092

[ref20] Hugelshofer C. L.; Magauer T. Nat. Prod. Rep. 2017, 34, 228–234. 10.1039/C7NP00005G. 28180228

[ref21] Wu X.; Sudhakar H. K.; Alcock L. J.; Lau Y. H. J. Med. Chem. 2023, 66, 11271–11281. 10.1021/acs.jmedchem.3c00674. 37555818

