# Supplementary material for: Novel Skeletal Rearrangements of the Tigliane Diterpenoid Core
Source: J Nat Prod. 2023 Nov 22;86(12):2685–90. doi: 10.1021/acs.jnatprod.3c00834 (PMC10749460; doi:10.1021/acs.jnatprod.3c00834)
Supplement: Supplementary file 1 — np3c00834_si_001.pdf [file np3c00834_si_001.pdf]

SUPPORTING MATERIAL

Novel Skeletal Rearrangements of the Tigliane Diterpenoid Core

Chiara Maioli[†], Hawraz Ibrahim M. Amin[†], Giuseppina Chianese[¥], Alberto Minassi[†], Paul W. Reddell[‡], Simone Gaeta[†,‡,*], Orazio Taglialatela-Scafati,[¥,*] and Giovanni Appendino[†]

[†]Dipartimento di Scienze del Farmaco, Largo Donegani 2, 28100 Novara, Italy
[¥]Dipartimento di Farmacia, Università di Napoli Federico II, Via Montesano 49, 80131 Napoli, Italy

[‡]QBiotics Group Limited, 165, Moggill Road, 4068, Taringa, Brisbane QLD, Australia

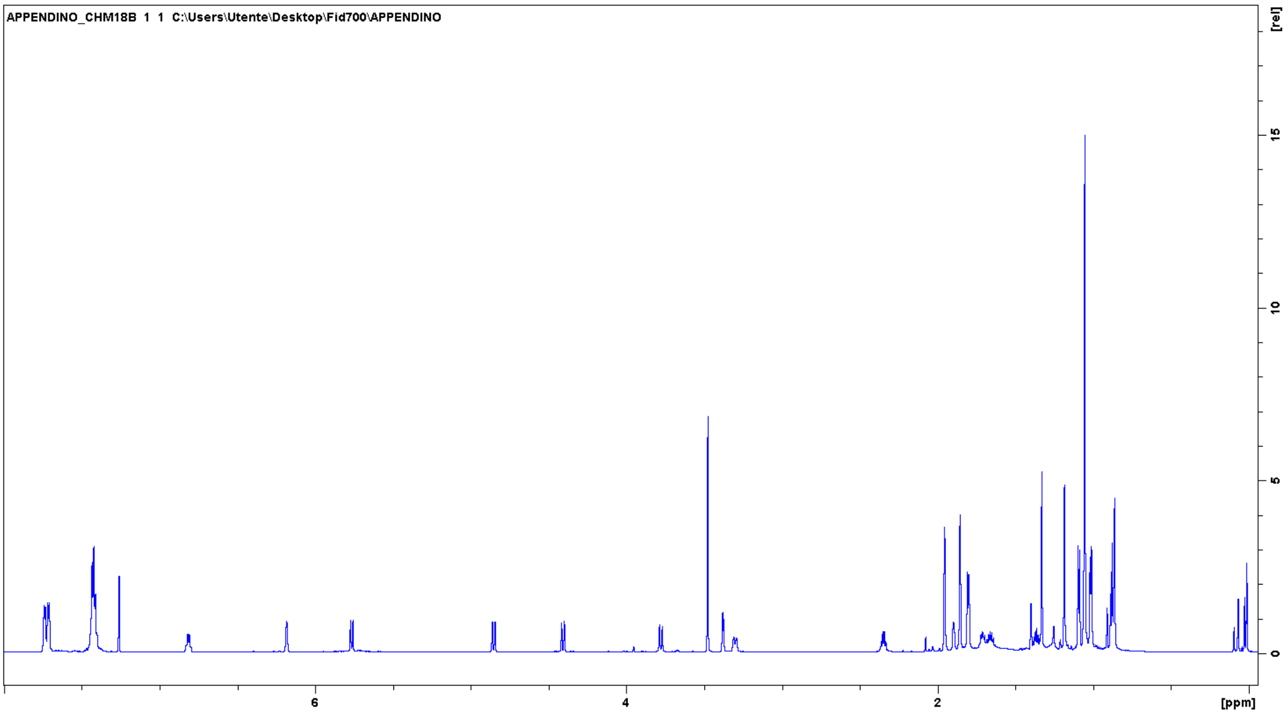

Figure S1. ^{1}H NMR spectrum of compound **4a** (700 MHz) in $CDCl_3$

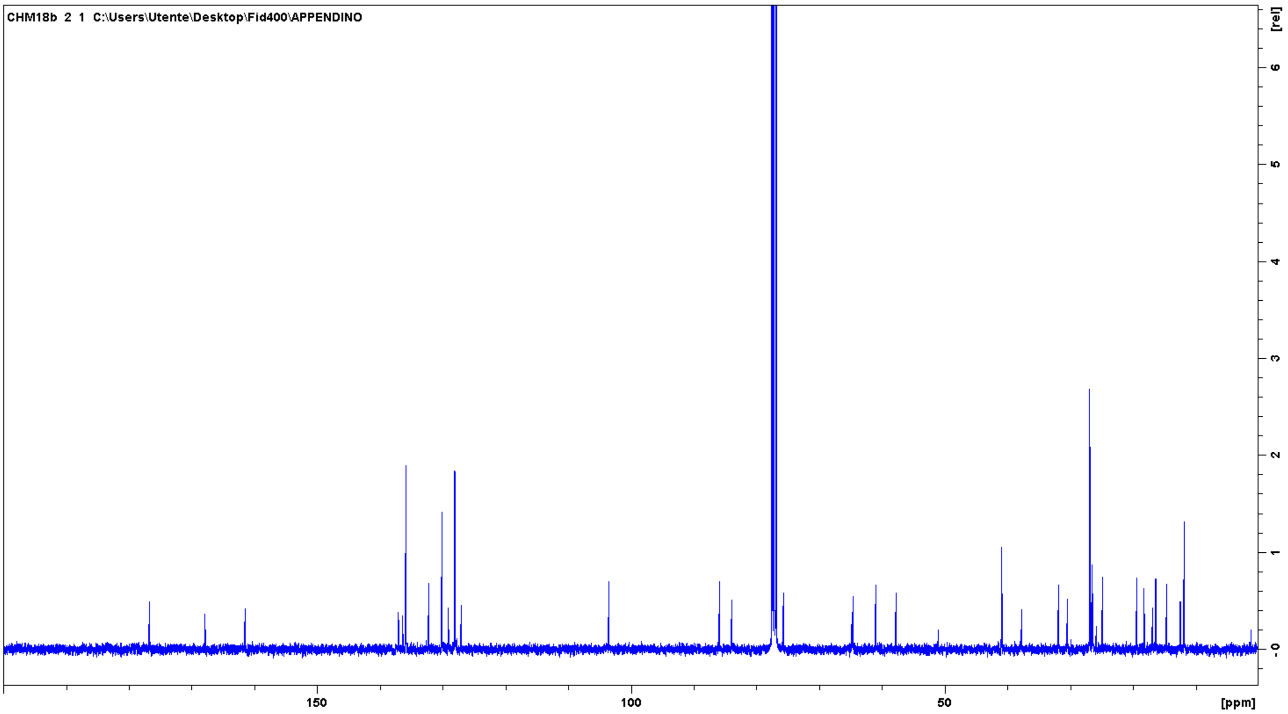

Figure S2. ^{13}C NMR spectrum of compound **4a** (400 MHz) in $CDCl_3$

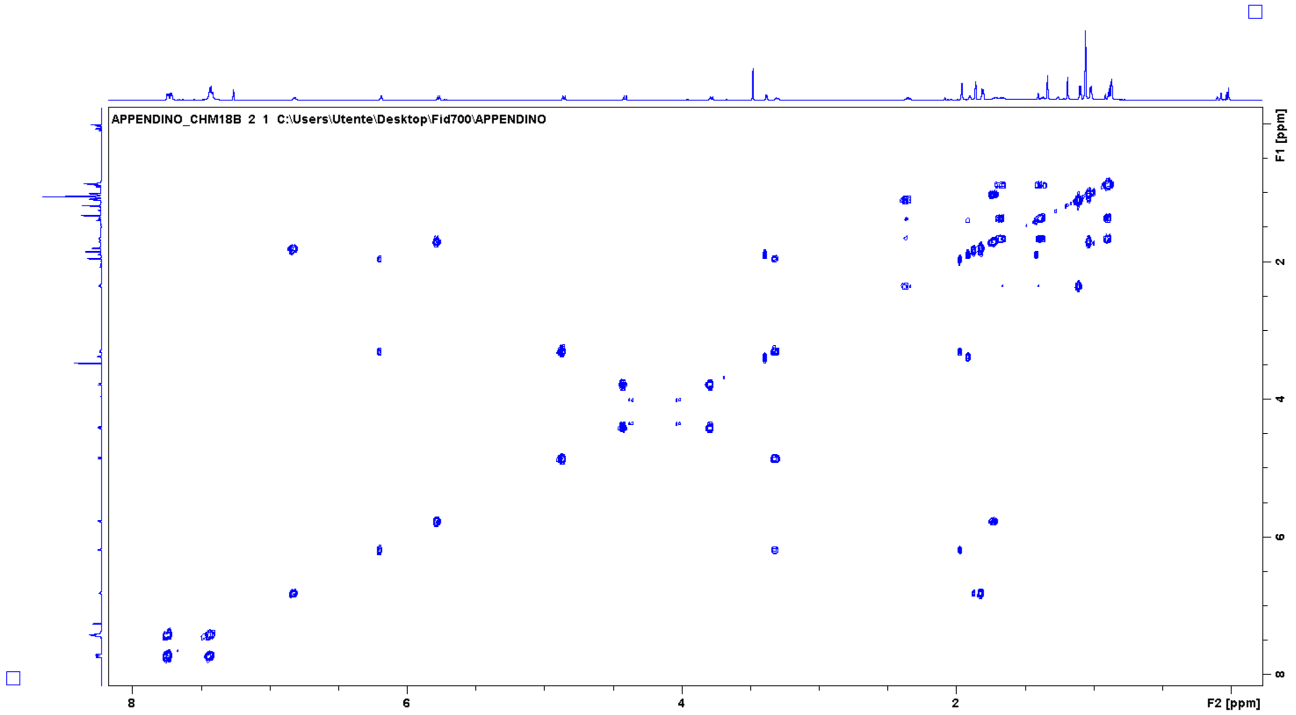

Figure S3. 2D NMR COSY spectrum of compound **4a** (700 MHz) in $CDCl_3$

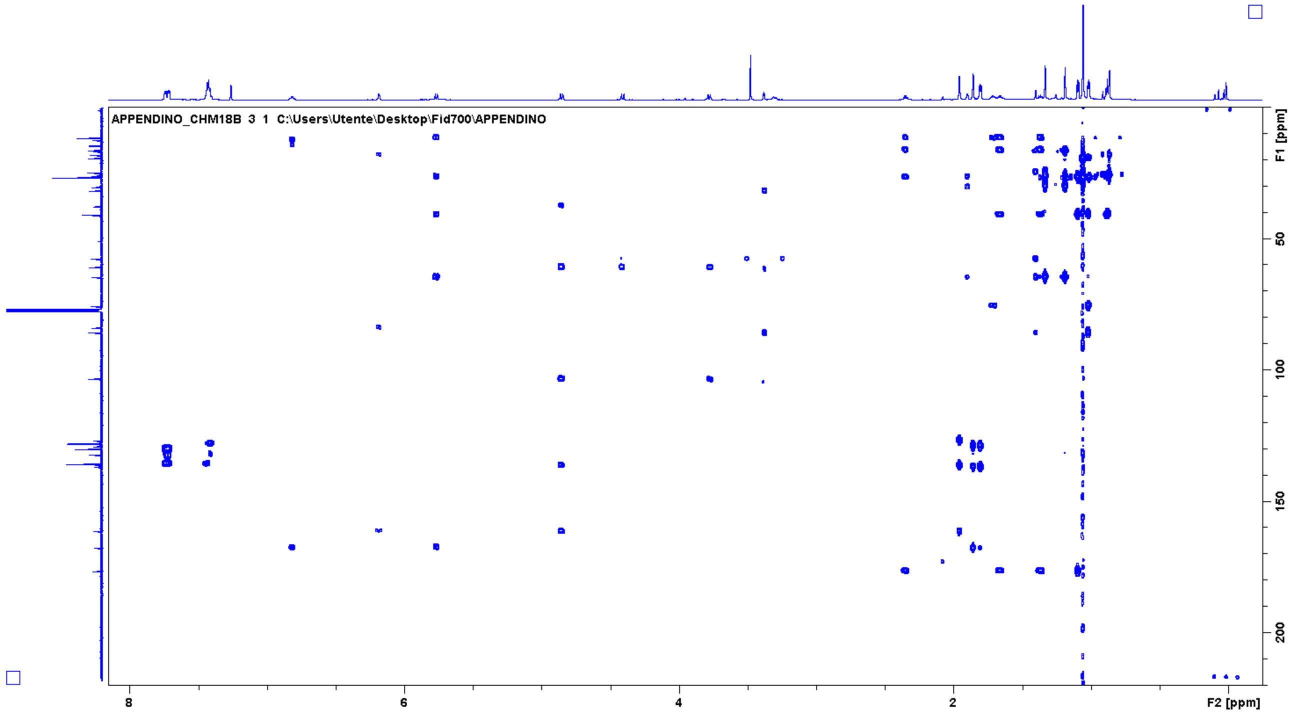

Figure S4. 2D NMR HMBC spectrum of compound **4a** (700 MHz) in $CDCl_3$

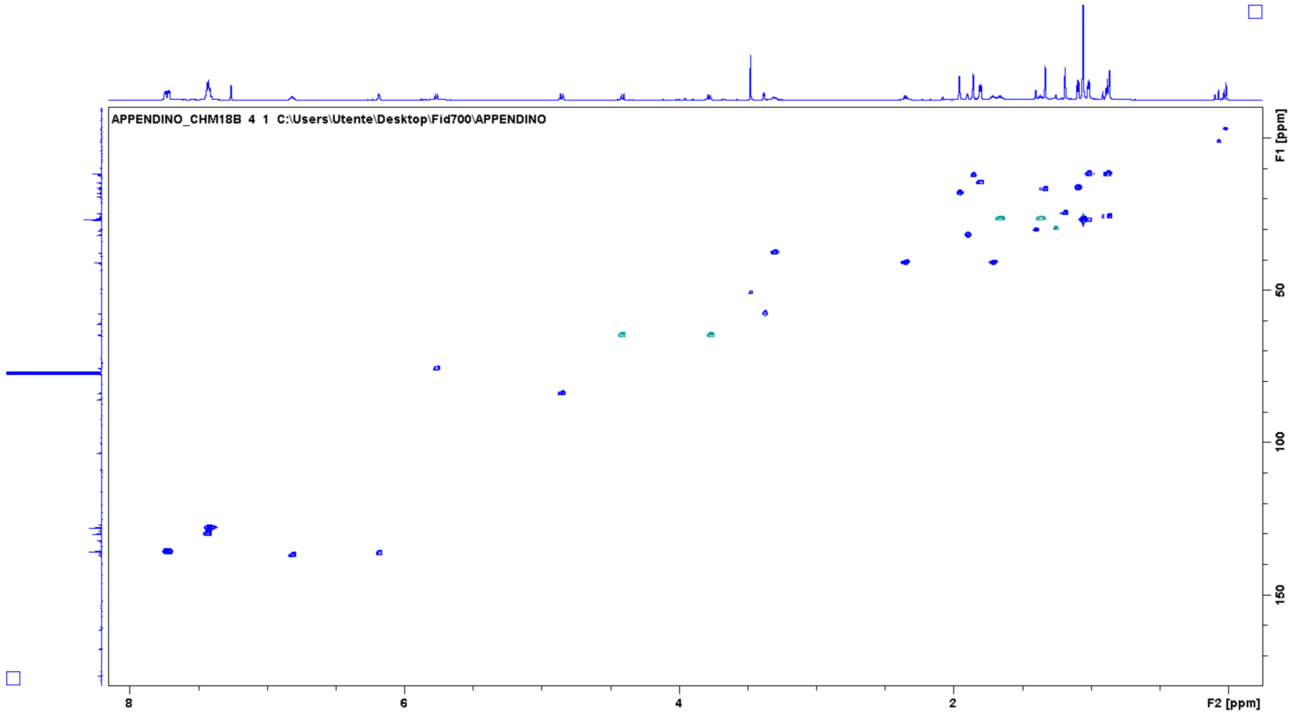

Figure S5. 2D NMR HSQC spectrum of compound **4a** (700 MHz) in $CDCl_3$

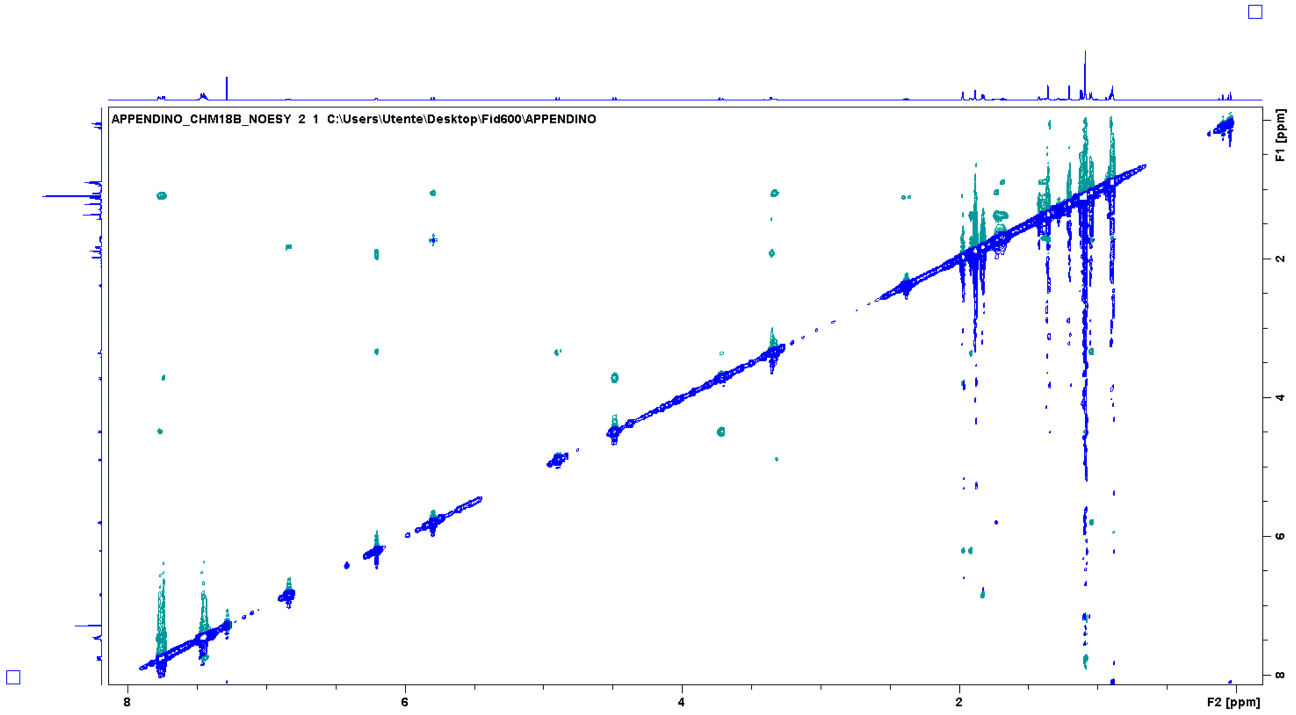

Figure S6. 2D NMR NOESY spectrum of compound **4a** (600 MHz) in $CDCl_3$

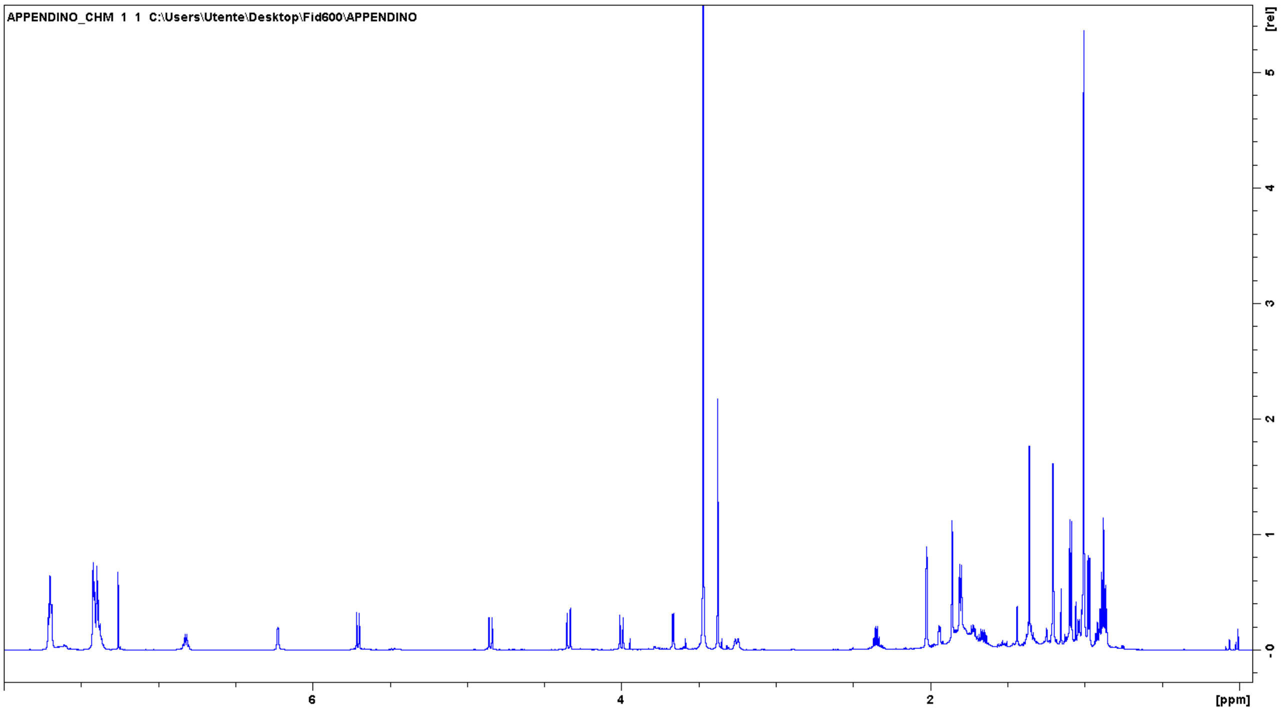

Figure S6. ^1H NMR spectrum of compound **4b** (600 MHz) in $CDCl_3$

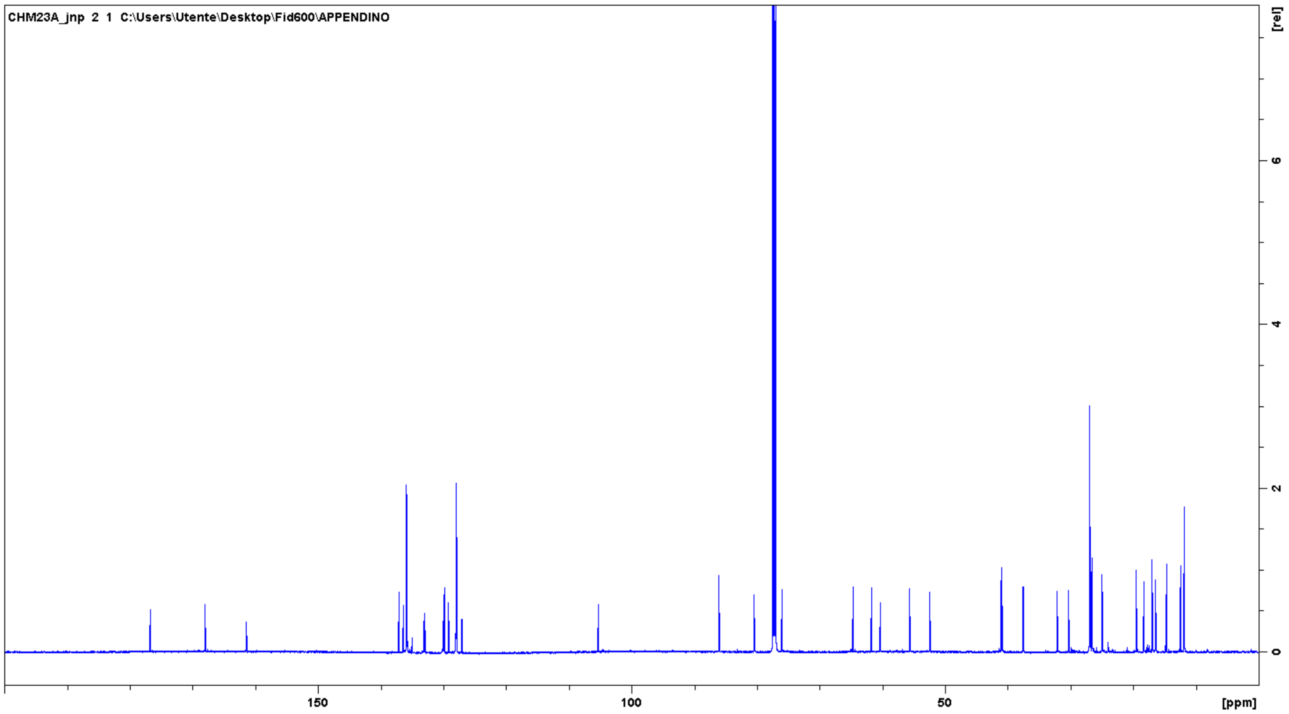

Figure S7. ^{13}C NMR spectrum of compound **4b** (600 MHz) in $CDCl_3$

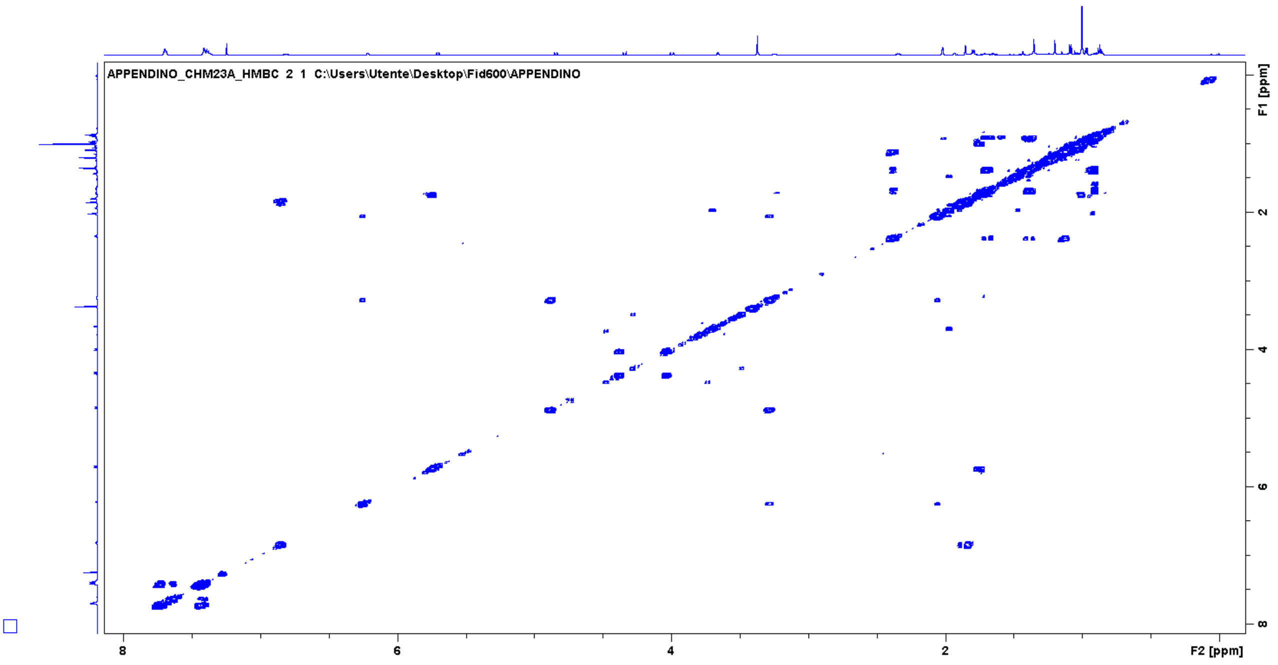

Figure S8. 2D NMR COSY spectrum of compound **4b** (600 MHz) in $CDCl_3$

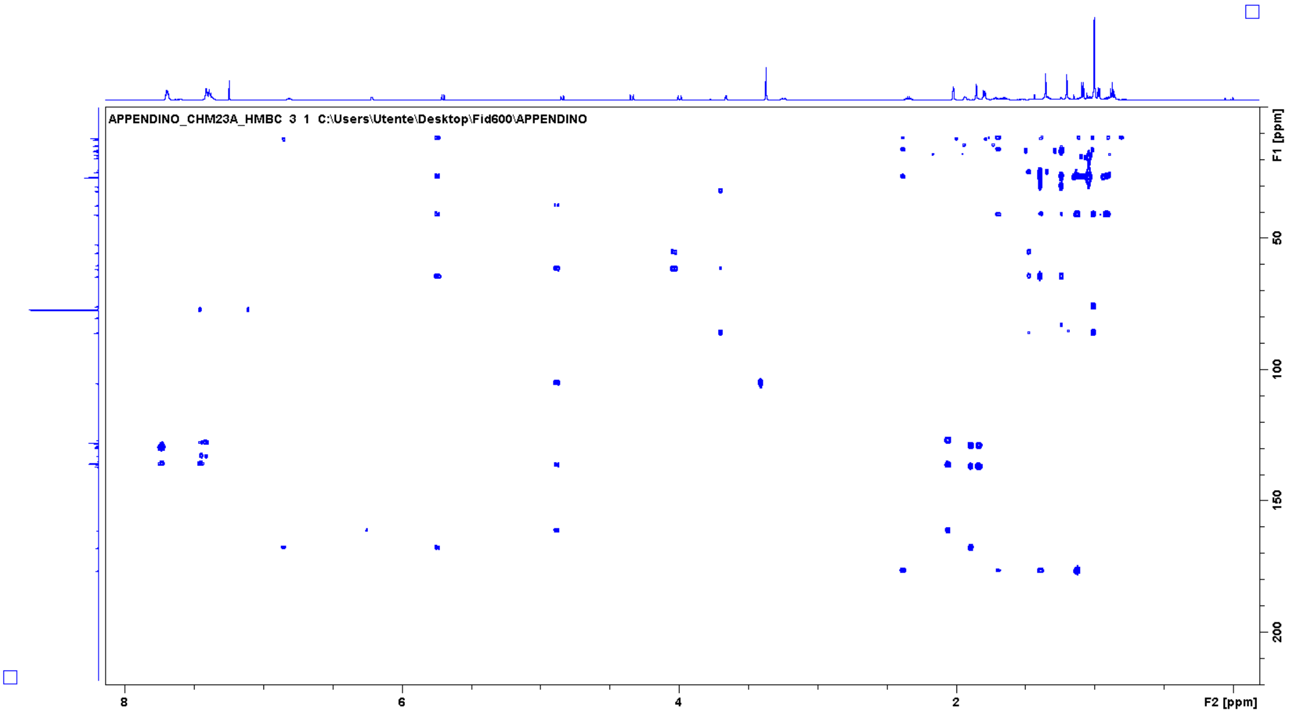

Figure S9. 2D NMR HMBC spectrum of compound **4b** (600 MHz) in $CDCl_3$

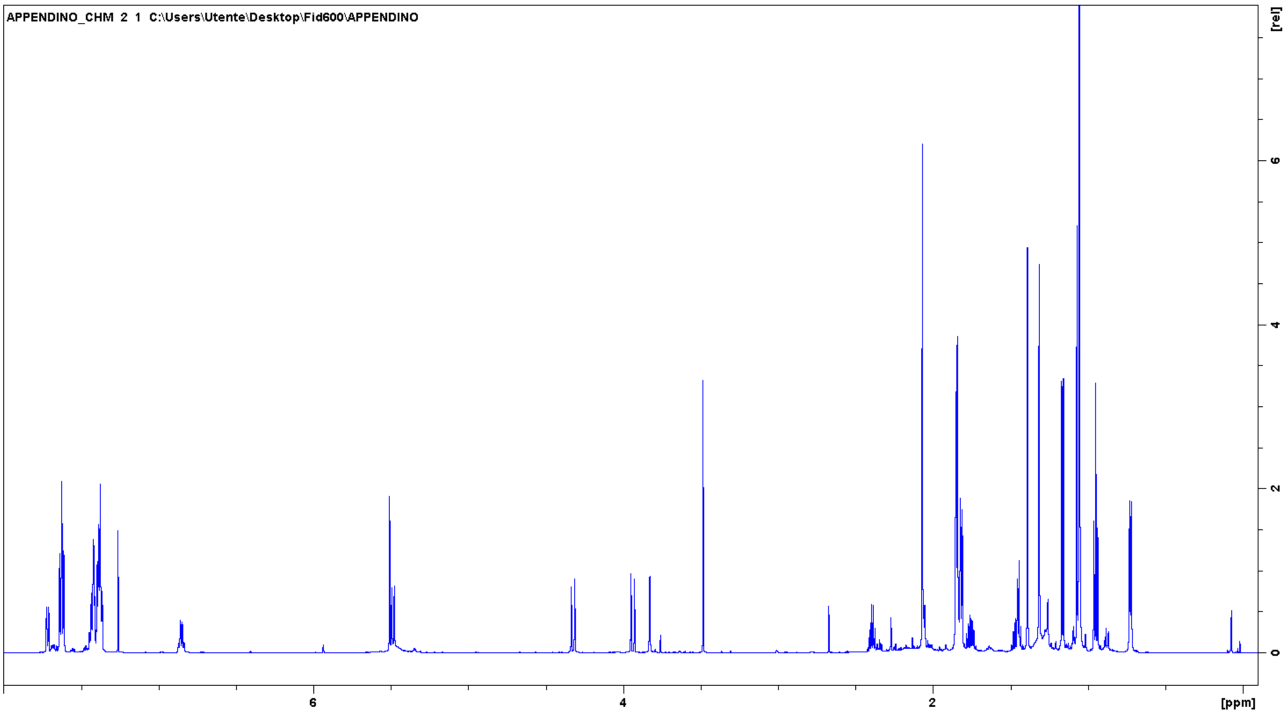

Figure S10. ^{1}H NMR spectrum of compound **5** (600 MHz) in $CDCl_3$

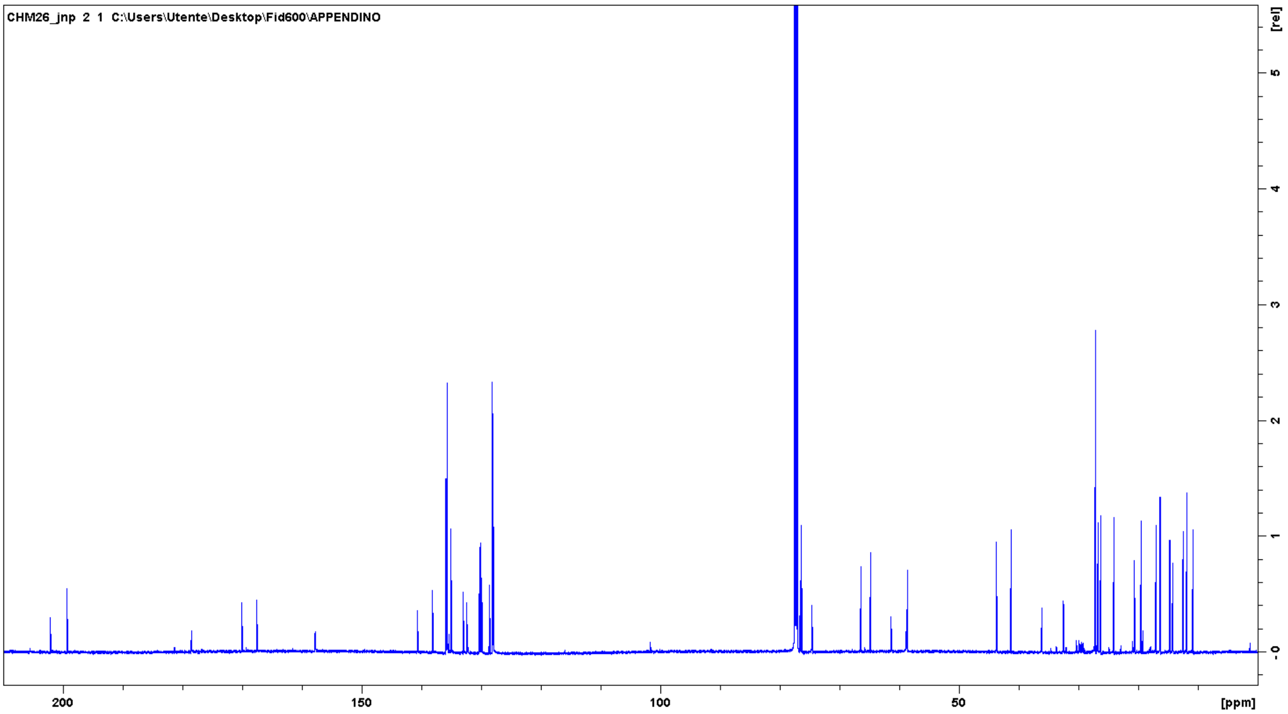

Figure S11. ^{13}C NMR spectrum of compound **5** (600 MHz) in $CDCl_3$

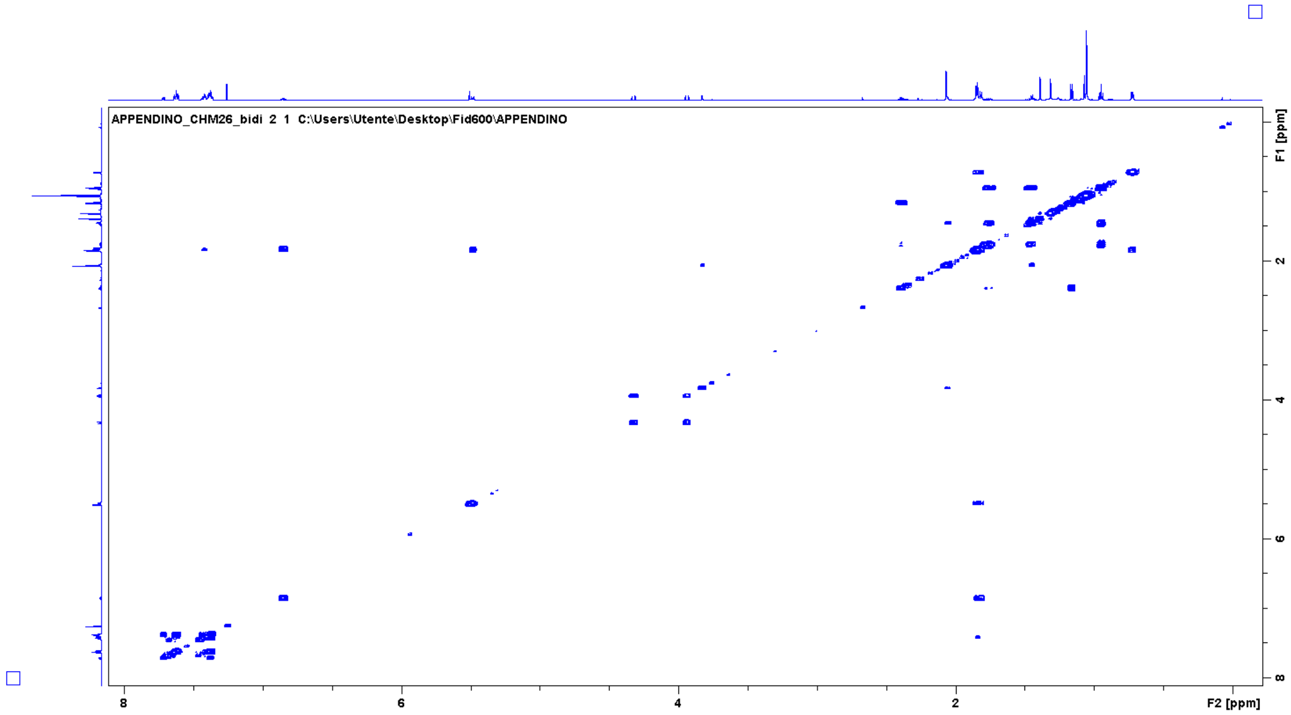

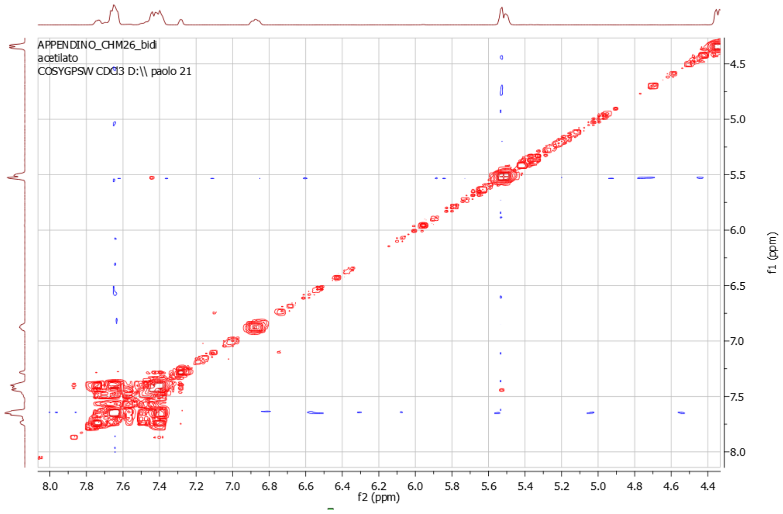

Figure S12. 2D NMR COSY spectrum of compound **5** (600 MHz) in $CDCl_3$ (top) and magnification of a region of the spectrum (bottom)

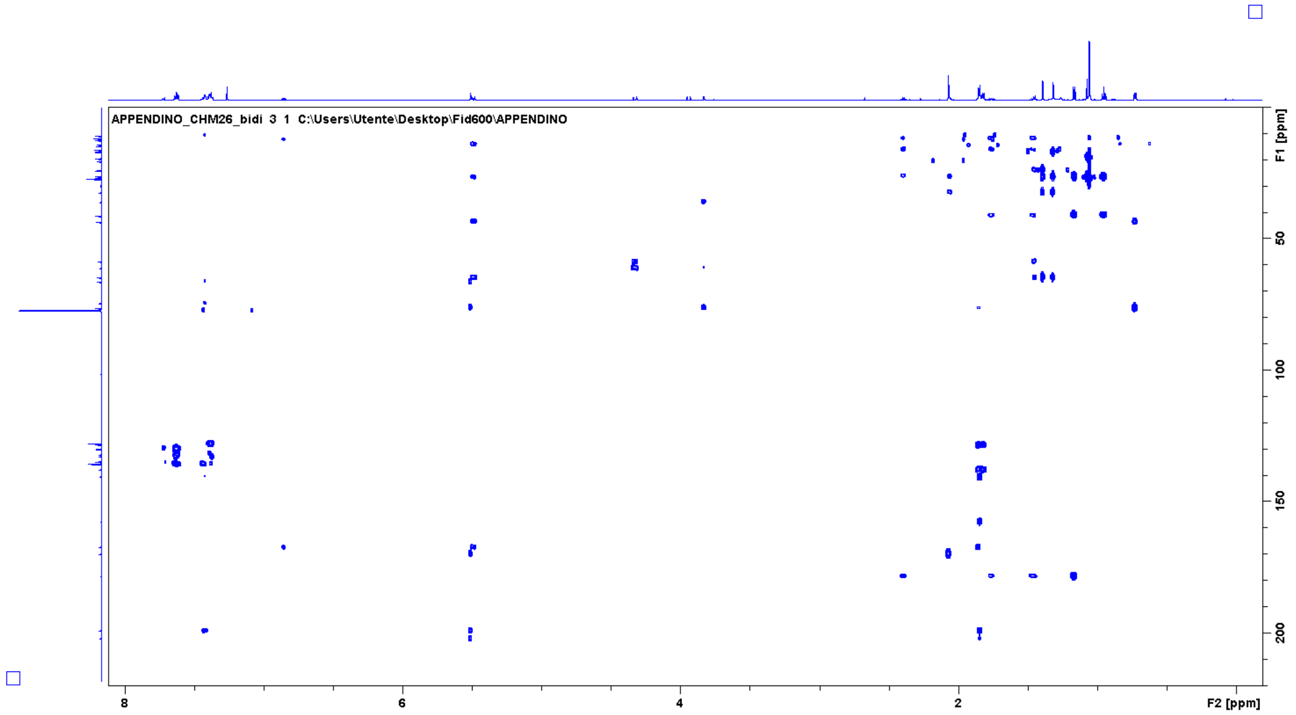

Figure S13. 2D NMR HMBC spectrum of compound **5** (600 MHz) in $CDCl_3$

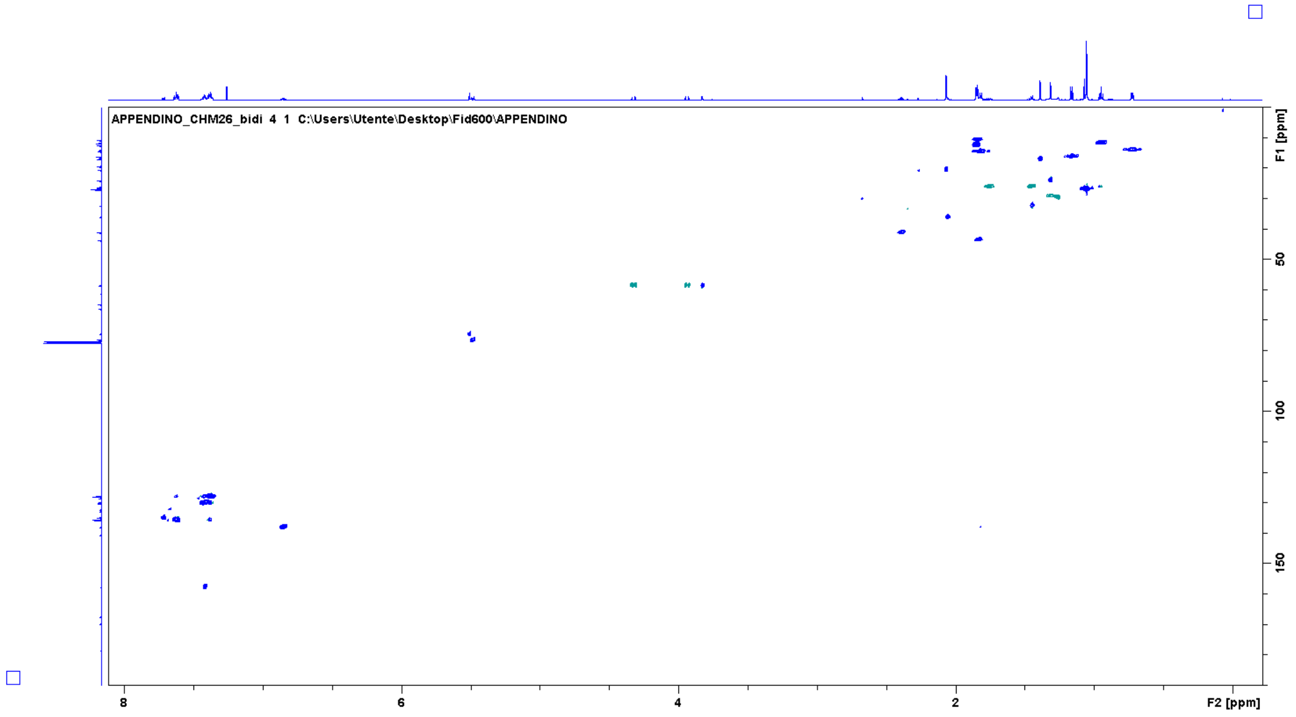

Figure S14. 2D NMR HSQC spectrum of compound **5** (600 MHz) in $CDCl_3$

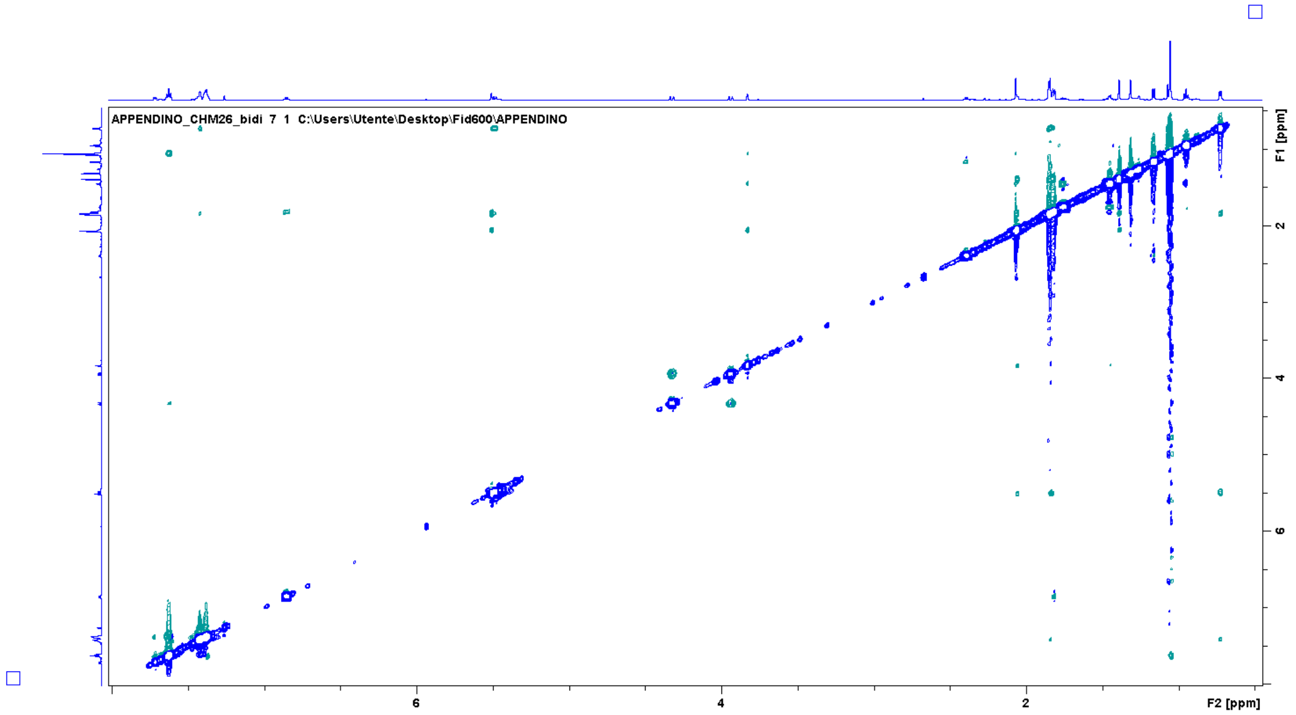

Figure S15. 2D NMR NOESY spectrum of compound **5** (600 MHz) in $CDCl_3$

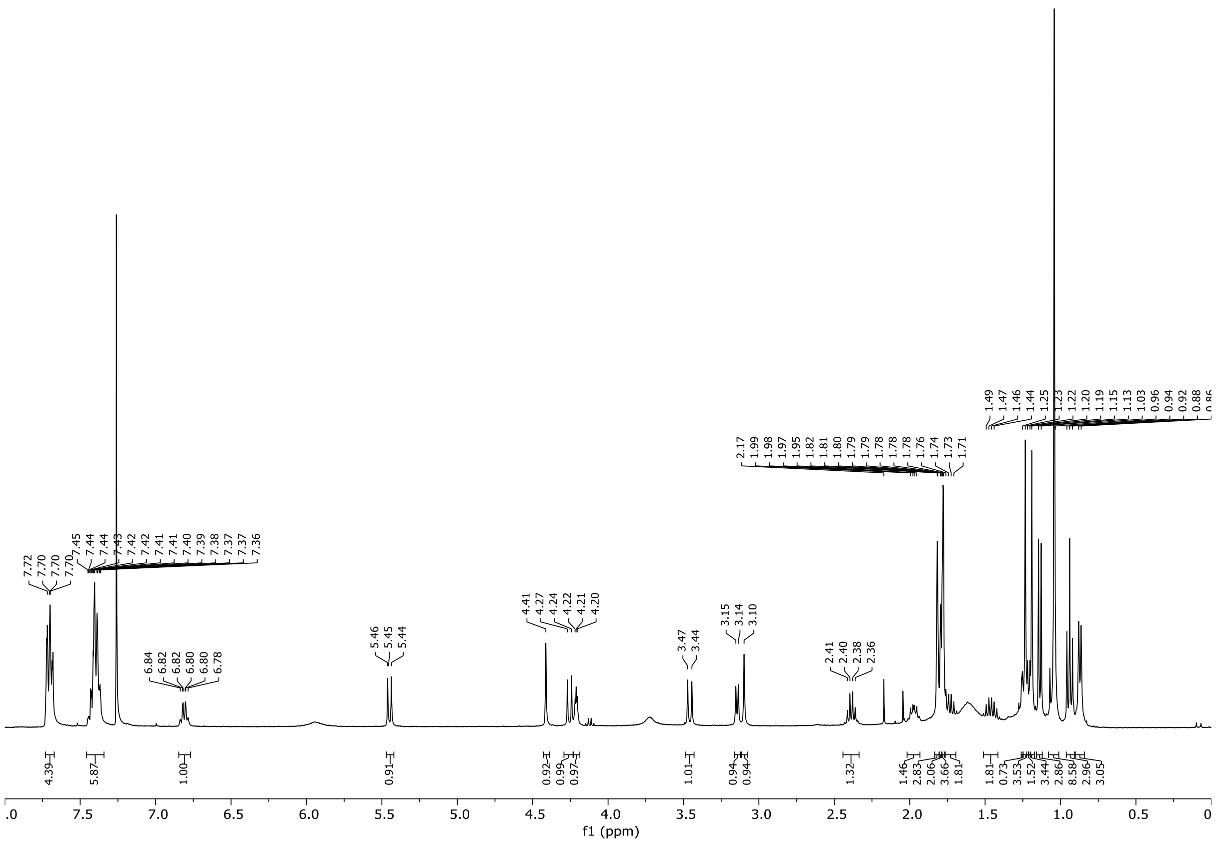

Figure S16. ^{1}H NMR spectrum of compound **2c** (400 MHz) in $CDCl_3$

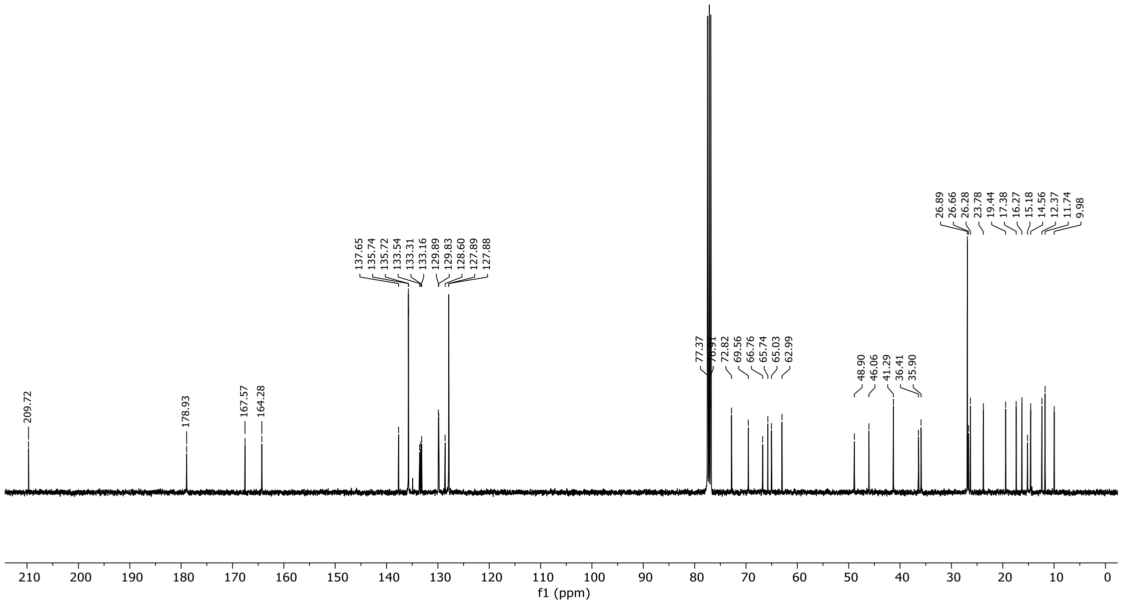

Figure S17. ^{13}C NMR spectrum of compound **2c** (100 MHz) in $CDCl_3$

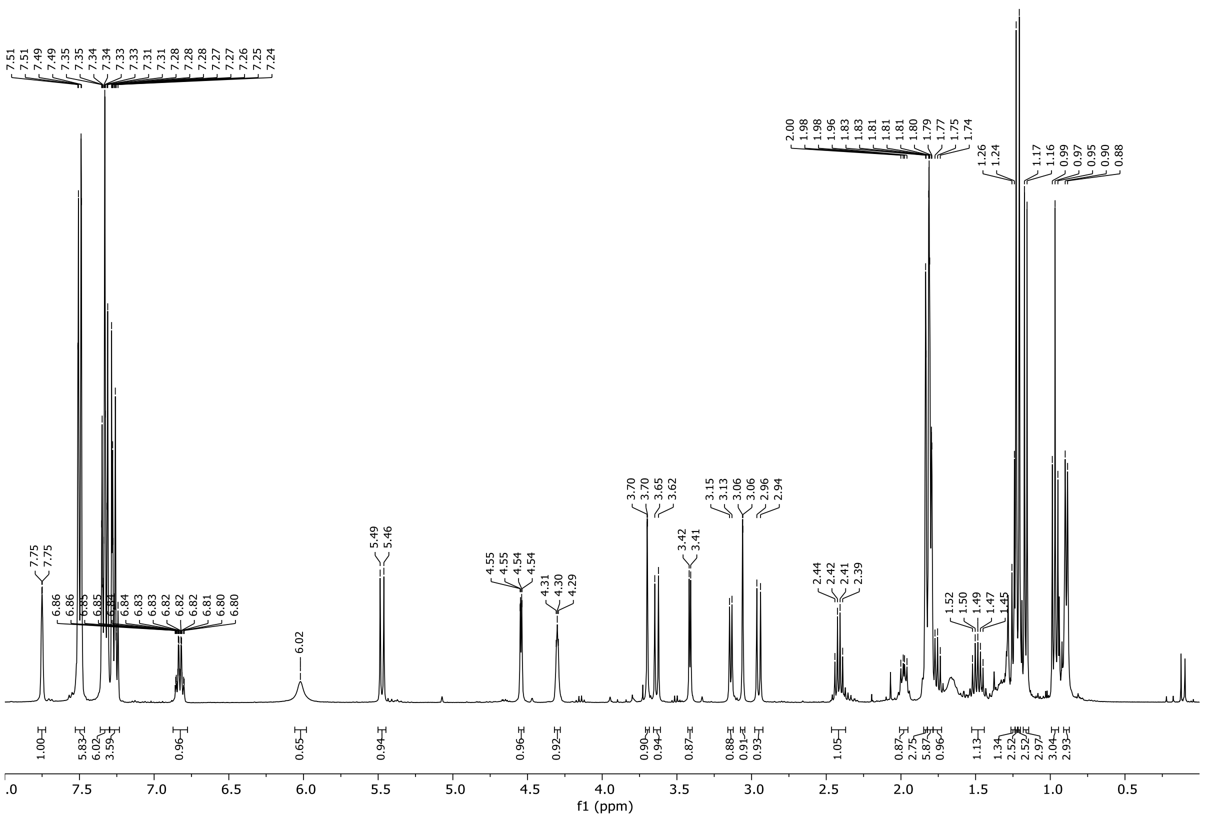

Figure S18. ^{1}H NMR spectrum of compound **2d** (400 MHz) in $CDCl_3$

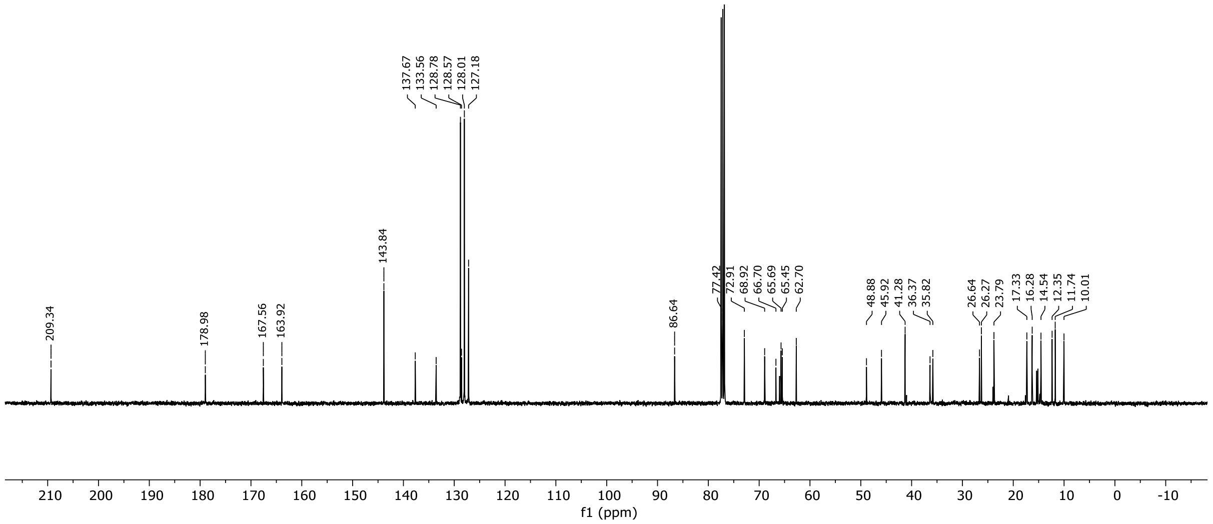

Figure S19. ^{13}C NMR spectrum of compound **2d** (100 MHz) in $CDCl_3$
